# Disrupting the DREAM complex enables proliferation of adult human pancreatic **β** cells

**DOI:** 10.1172/JCI157086

**Published:** 2022-08-01

**Authors:** Peng Wang, Esra Karakose, Carmen Argmann, Huan Wang, Metodi Balev, Rachel I. Brody, Hembly G. Rivas, Xinyue Liu, Olivia Wood, Hongtao Liu, Lauryn Choleva, Dan Hasson, Emily Bernstein, Joao A. Paulo, Donald K. Scott, Luca Lambertini, James A. DeCaprio, Andrew F. Stewart

**Affiliations:** 1 Diabetes Obesity Metabolism Institute,; 2 Department of Medicine, and; 3 Department of Genetics and Genomic Sciences, The Icahn School of Medicine at Mount Sinai, New York, New York, USA.; 4 Sema4, Stamford, Connecticut, USA.; 5 Department of Pathology, The Icahn School of Medicine at Mount Sinai, New York, New York, USA.; 6 Dana-Farber Cancer Institute, Boston, Massachusetts, USA.; 7 The Department of Medicine, Brigham and Women’s Hospital, Harvard Medical School, Boston, Massachusetts, USA.; 8 Department of Pediatrics,; 9 The Tisch Cancer Institute,; 10 Department of Oncological Sciences,; 11 Bioinformatics for Next Generation Sequencing (BiNGS) Shared Resource Facility, and; 12 The Graduate School of Biomedical Sciences, The Icahn School of Medicine at Mount Sinai, New York, New York, USA.

**Keywords:** Endocrinology, Beta cells, Diabetes

## Abstract

Resistance to regeneration of insulin-producing pancreatic β cells is a fundamental challenge for type 1 and type 2 diabetes. Recently, small molecule inhibitors of the kinase DYRK1A have proven effective in inducing adult human β cells to proliferate, but their detailed mechanism of action is incompletely understood. We interrogated our human insulinoma and β cell transcriptomic databases seeking to understand why β cells in insulinomas proliferate, while normal β cells do not. This search reveals the DREAM complex as a central regulator of quiescence in human β cells. The DREAM complex consists of a module of transcriptionally repressive proteins that assemble in response to DYRK1A kinase activity, thereby inducing and maintaining cellular quiescence. In the absence of DYRK1A, DREAM subunits reassemble into the pro-proliferative MMB complex. Here, we demonstrate that small molecule DYRK1A inhibitors induce human β cells to replicate by converting the repressive DREAM complex to its pro-proliferative MMB conformation.

## Graphical abstract

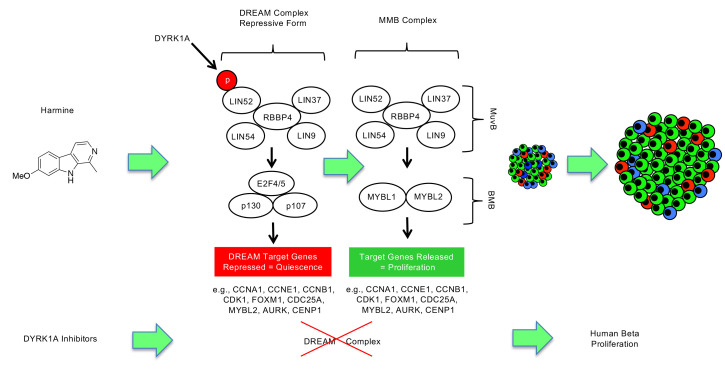

## Introduction

Type 1 (T1D) and type 2 (T2D) diabetes together afflict 463 million people globally (1). While T1D results from autoimmune destruction of pancreatic insulin-producing β cells, and T2D is widely perceived to result from resistance to insulin in liver, skeletal muscle, and adipose tissue, the 2 conditions share at least 2 important features. First, both T1D and T2D are associated with marked reductions in the numbers of insulin-producing pancreatic β cells (2–6). Second, almost everyone with T1D and T2D has at least some viable residual pancreatic β cells (2–6). The reduction in β cell numbers has prompted attempts to replace missing β cells by whole pancreas transplant, by transplant of isolated pancreatic islets from organ donors, or by transplant of β cells derived from human stem cells. Each of these approaches has made remarkable progress over the past 2 decades, yet none is scalable to millions of people with T1D and T2D for reasons of cost and donor organ availability. These considerations have prompted searches for pharmacologic approaches to induce regeneration and/or redifferentiation of the β cells that remain in people with diabetes, to repopulate the pancreas, and to do so in a manner that is scalable to the hundreds of millions of people afflicted with diabetes.

Recently, several groups have demonstrated that small molecule drugs that inhibit the kinase DYRK1A (dual tyrosine–regulated kinase 1A) are able to induce adult human β cells to proliferate, to increase in numbers, and to enhance their differentiation and function (7–17). Even higher rates of proliferation can be achieved by combining DYRK1A inhibitors with peptide agonists of the GLP1 receptor such the GLP1_7–36_ peptide, or more stable synthetic analogs, such as exendin-4, liraglutide, semaglutide, and others, all in current widespread use in T2D (10, 12). The combination of a DYRK1A inhibitor, harmine, together with exendin-4, increases human β cell mass in immunodeficient mice transplanted with human islets by 700% over 3 months of treatment, while also reversing diabetes (12).

**Figure 1:**
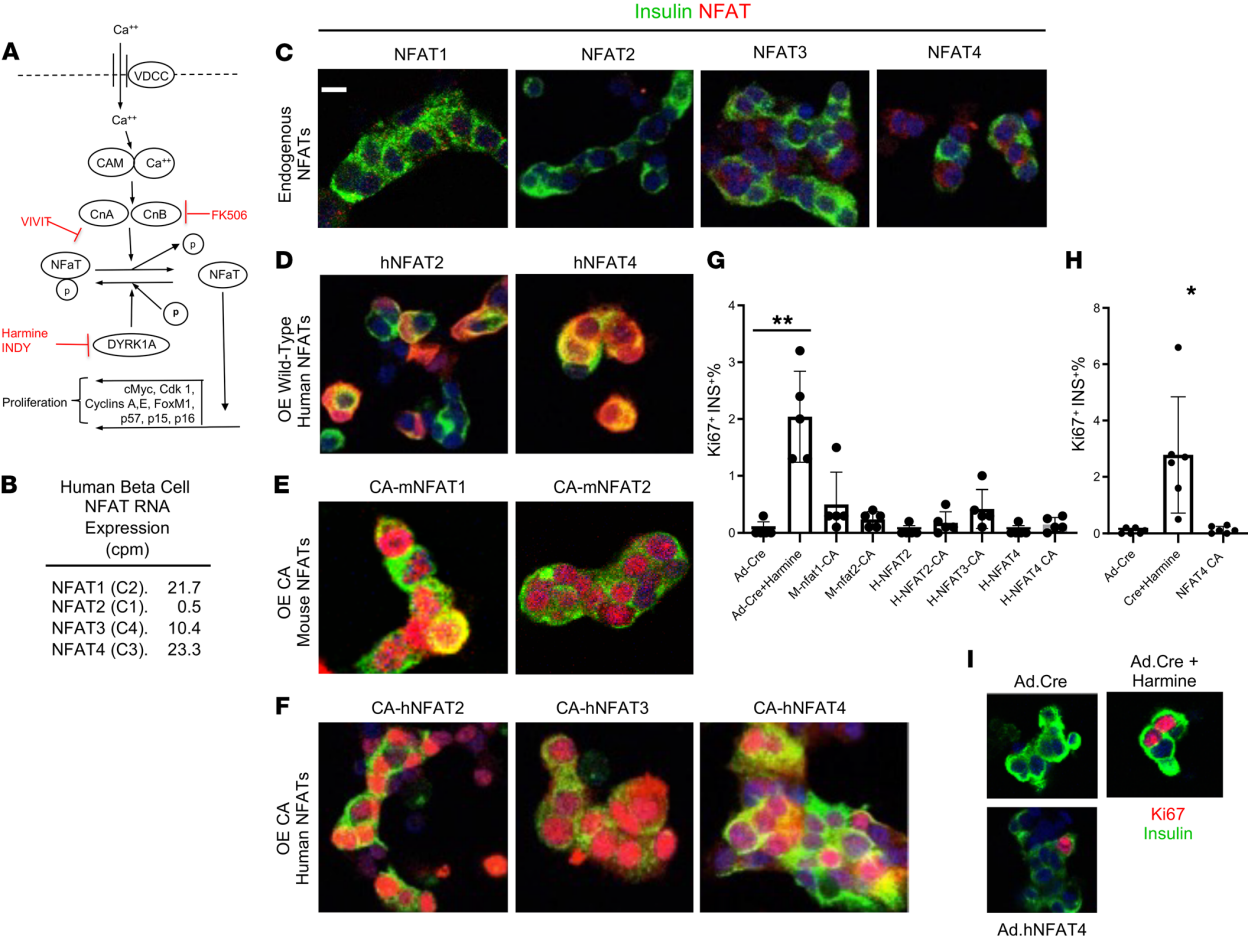
NFAT expression and overexpression in human β cells. (**A**) The current prevailing mechanism of action model for the proliferative effects of DYRK1A inhibition. Cam, calmodulin; VDCC, voltage-dependent calcium channel. VIVIT is a small peptide NFAT-calcineurin inhibitor, FK506 is a calcineurin inhibitor. Harmine and INDY are small molecule inhibitors of DYRK1A. See main text for complete details. (**B**) Expression levels of the 4 human NFATs in RNA-seq from 22 sets of FACS-isolated human β cells, from Wang et al. (46). See Results for explanation of NFAT nomenclature. Values are in counts per million reads (CPM). (**C**) Expression of endogenous NFATs in human β cells assessed by immunohistochemistry. All are expressed, but only at low levels. (**D**) Overexpression by CMV promoter–driven adenovirus of wild-type human NFAT2 and -4. Note that these NFATs are predominantly cytoplasmic in human β cells. (**E**) Overexpression of constitutively active mouse NFAT1 and -2 in human islets, as detected with antibodies against NFAT1 and NFAT2. (**F**) Overexpression of constitutively active human NFAT2, -3, and -4. Note that in contrast to panels C and D, these are predominantly nuclear, as anticipated. Panels C–E are representative of 3 different human islet preparations. Here again, NFAT expression is predominantly nuclear in β cells, as anticipated. (**G** and **H**) Effect of overexpression of wild-type and constitutively active NFATs on Ki67 immunolabeling in human β cells in islets from 5 (**G**) or 6 (**H**) different organ donors, compared to the DYRK1A inhibitor harmine, a positive control, and to the negative control, an adenovirus expressing Cre recombinase, all at the same MOI as the NFATs. Data are presented as mean ± SEM. 2-tailed Student’s paired *t* test, *P < 0.05; **P < 0.01. (**I**) Examples of Ki67 immunolabeling in human islets under the conditions shown. Scale bars: 10 μm.

The current understanding of the DYRK1A inhibitor mechanism of action in pancreatic β cells is derived from earlier experiments in T lymphocytes as well as rodent and human β cells (Figure 1) (7–28). This model suggests that pro-proliferative signals to β cells, exemplified by calcium entry, sequentially activate calmodulin and calcineurin. Calcineurin then dephosphorylates the cytoplasmic family of NFAT (nuclear factors in activated T cells) transcription factors, allowing them to translocate to the nucleus where they transactivate genes encoding cell cycle activators, exemplified by D- and A-cyclins, *CDK1*, and *FOXM1*, while repressing genes encoding cell cycle inhibitors, such as *CDKN1A* (encoding p21^CIP1^), *CDKN1C* (encoding p57^KIP2^), *CDKN2A* (encoding p16^INK4^), and *CDKN2B* (encoding p15^INK4^), with the effect of driving entry into the cell cycle and progression through S and G_2_M phases. In this scenario, DYRK1A serves as a nuclear kinase that rephosphorylates NFAT transcription factors, thereby expelling them from the nucleus, and terminating their mitogenic effects. Thus, in this paradigm, NFATs are the activators of β cell proliferation, and DYRK1A is the “brake” on human β cell proliferation.

This NFAT-driving proliferation scenario is supported in β cells by reports from several laboratories showing that DYRK1A inhibitors induce rodent and human β cells to replicate, that NFATs translocate to the nucleus in response to intracellular calcium increments, and that they bind to promoters of cell cycle regulatory genes (7–17). On the other hand, calcineurin inhibitors, such as FK506 and the short peptide VIVIT (Figure 1), only partially attenuate, and do not eliminate, human β cell proliferation induced by DYRK1A inhibition (8, 16). Thus, the final leg of the pathway, the NFAT-to-proliferation leg, merits deeper exploration. Accordingly, here we sought to determine whether overexpression of wild-type or constitutively active NFAT isoforms is capable of driving adult human β cells to proliferate. To our surprise, none of the 4 NFAT isoforms induced proliferation in human β cells to a degree comparable to that induced by harmine. This led us to explore alternate mitogenic pathways downstream of DYRK1A inhibition. This search revealed the central importance of the DREAM complex in enforcing quiescence in adult human β cells.

**Figure 2:**
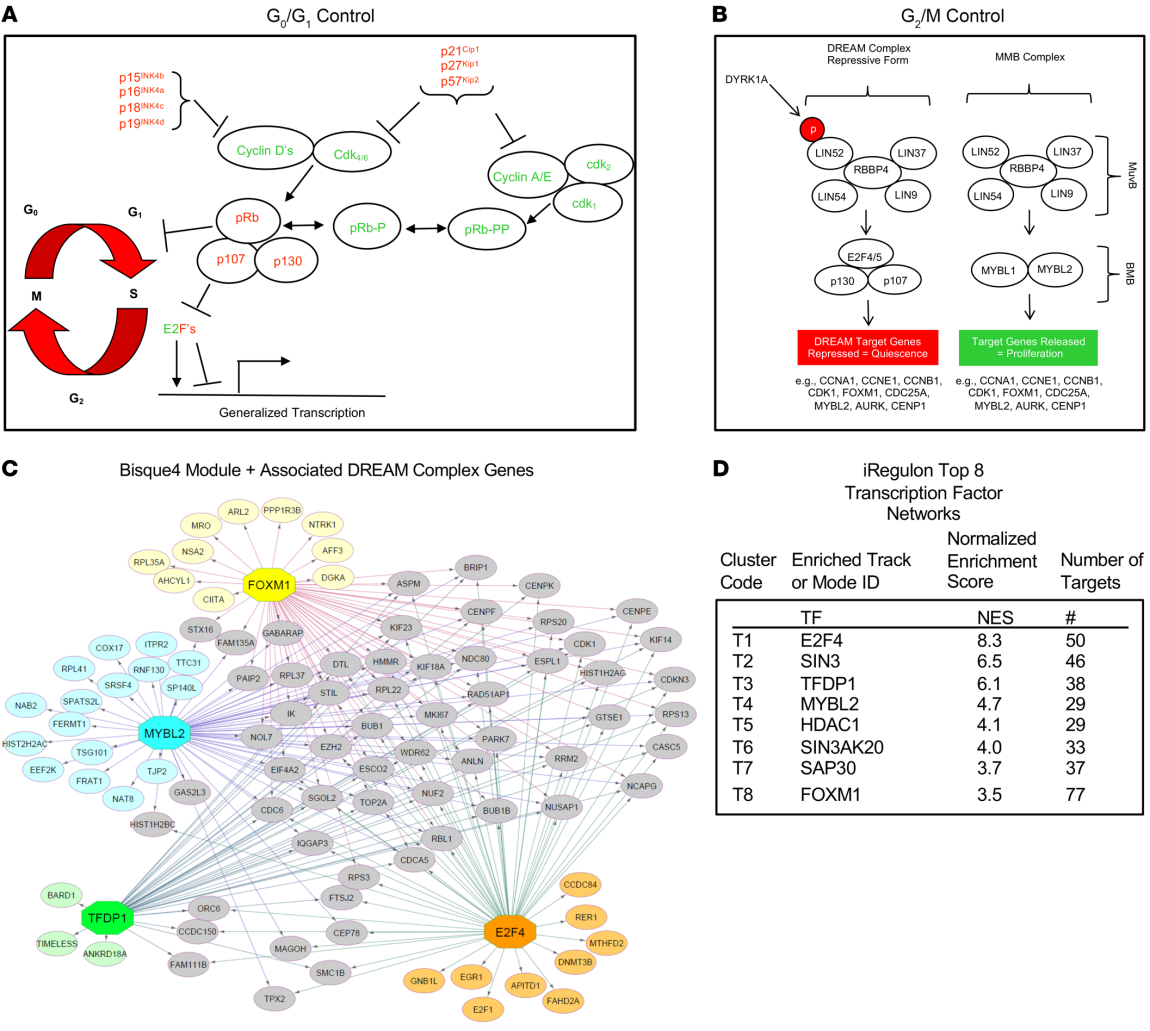
Conventional cell cycle control, DREAM complex anatomy, and human insulinoma bioinformatics. (**A**) Conventional model of cell cycle molecules that regulate transition from G_0_ into G_1_ and S phases of the mammalian cell cycle (see refs. 32, 33 for reviews). (**B**) The 2 configurations of the mammalian DREAM and MMB complexes (see the Introduction and refs. 38–46 for details). (**C**) The predicted targets in the Bisque4 module membership group of these transcriptional regulators are shown as a network. An enlarged version is shown in Supplemental Figure 3. Nodes are colored according to the predicted transcriptional regulator they are in the network (yellow = FOXM1; cyan = MYBL2; green = TFDP1; orange = E2F4). Genes (nodes) that have edges/connections coming from multiple transcription factors (TFs) are colored gray. See main text for details. (**D**) The iRegulon tool was used to explore the predicted upstream transcriptional regulators of the Bisque4 module membership group of 253 genes derived from the WGCNA of a cohort of human insulinomas (Supplemental Figure 2) (46). The top TFs predicted by iRegulon, namely, E2F4, TFDP1, MYBL2, and FOXM1, are all canonical DREAM complex members (see Supplemental Table 3 for full results). For the normalized enrichment score, anything above 3.0 was considered significant. “# targets” indicate the number of genes predicted as targets of that TF that were found in the gene set of interest. Databases associated with binding motifs (M) or Encode ChIP-seq tracks (T) were surveyed within iRegulon.

Mammalian cell cycle entry is initiated by interactions among canonical members of the G_1_/S pathway (Figure 2A). The retinoblastoma protein (RB) enforces arrest in G_0_/G_1_, but can be inactivated through phosphorylation by CDK4 or CDK6 acting in concert with D-cyclins. RB can be further phosphorylated and inactivated by CDK1 and CDK2 in concert with A- or E-cyclins, events that lead to G_1_/S passage. Upstream of these cyclins and CDKs, CDK inhibitors such as p16^INK4A^, p15^INK4B^, p18^INK4C^, p19^INK4D^, p21^CIP1^, p27^CIP2^, and p57^KIP2^ block the activities of cyclin-CDK complexes and prevent G_1_/S entry. Detailed summaries of these events in β cells are available (29–37). In contrast to G_1_/S, transition into and through G_2_M is largely controlled by the DREAM complex, which exists in 2 formats (Figure 2B and Table 1), as detailed in several recent reviews (38–45). In its repressive format, a central core of MuvB proteins (consisting of LIN52, LIN9, LIN37, LIN54, and RBBP4) are recruited to the cell cycle inhibitor, p130, and its partners E2F4 (or E2F5) and DP1 (gene name *TFDP1*). This cluster of proteins assembles on consensus DP1, E2F4, p130 binding sites (CDEs) of, and thereby repressing, some 1000 target genes involved in G_2_M entry, progression, and other cellular activities (38–45). The key to forming and maintaining the repressive version of the DREAM complex is phosphorylation of LIN52 on Ser^28^, an event that leads to recruitment of MuvB proteins — consisting of LIN9, LIN37, LIN52, LIN54, and RBBP4 — to p130/E2F4/DP1 and formation of the repressive DREAM complex. This LIN52 Ser^28^ phosphorylation is performed by the kinase DYRK1A (38–45).

Upon entry into S phase, the DREAM complex assembles into an alternate, pro-proliferative configuration containing the MuvB complex and B-MYB (MYBL2) referred to as the “MMB complex” (Figure 2B) (38–45). Here, LIN52 Ser^28^ is not phosphorylated, with the result that the MuvB partners are not recruited to the repressive partners, p130, E2F4, and DP1. Instead, LIN52 and the other MuvB members recruit 2 pro-proliferative transcription factors, MYBL2 and FOXM1, to cell cycle homology regions of target genes, events that convert the DREAM complex from a repressive complex in G_0_ to the MMB-FOXM1 complex that favors G_2_M passage (38–45). Thus, DYRK1A serves as a switch that converts the pro-proliferative MMB configuration to the repressive DREAM configuration. It follows that small molecule DYRK1A inhibitors have the potential to convert quiescent cells to proliferating cells by converting the repressive DREAM complex to the proliferative MMB configuration.

Here, we report that DREAM plays a central role in enforcing replicative quiescence in the adult human β cell. We also report that small molecule DYRK1A inhibitors convert DREAM from its repressive configuration to the proliferative MMB conformation. This comprehensive model for control of cell cycle in the human β cell alters a well-established paradigm in the field of diabetes. Moreover, it provides a mechanism of action to explain how DYRK1A inhibitors induce human β cells to replicate. Finally, to the best of our knowledge it is the first time the biology of DREAM has been comprehensively defined in any nonmalignant human cell type.

## Results

### NFATs fail to activate human β cell proliferation.

NFATs exist in 4 classical forms, NFAT1, -2, -3, and -4, (also termed NFATC2, NFATC1, NFATC4, and NFATC3, respectively) (Figure 1B). While all are expressed at low levels, NFAT1, -3, and -4 are most abundant in human β cells (9, 26–28, 46–49), and are marginally detectable by immunohistochemistry on dispersed human islets (Figure 1, B and C) (7). NFATs have been shown repeatedly to translocate to the β cell nucleus in response to DYRK1A inhibition (8, 13, 15, 16, 18–23, 25, 26), but whether this directly leads to cell cycle entry is less clear. To assess the effects of NFATs on human β cell proliferation, we overexpressed wild-type NFAT2 or NFAT4 in dispersed human islets using adenoviruses driven by the CMV promoter. This led to dramatic increases in NFAT abundance in human β cells, but the 2 NFATs remained predominantly cytoplasmic (Figure 1D). We attributed this to a requirement for NFATs to be dephosphorylated to permit nuclear entry and retention (18–22). Two constitutively active (CA) mouse NFATs have been reported to drive human and rodent β cell replication, mNFATC1 and mNFATC2 (27, 28); thus, we also overexpressed these in human islets, and observed that they appeared strongly nuclear, as expected (Figure 1E). We also explored 3 different constitutively active CMV promoter–driven human NFATs, CA-hNFAT2, CA-hNFAT3, and CA-hNFAT4, which contain 8 to 21 serine-to-alanine substitutions (18–21). As expected, these 3 CA-hNFATs were highly expressed and were predominantly nuclear (Figure 1F). Collectively, these results align with the concept that NFATs are present in human β cells, and that they are predominantly cytoplasmic under basal conditions, but can translocate to the nucleus in response to DYRK1A inhibition and/or activation through dephosphorylation.

We next explored proliferation in human β cells, as detected by increases in the percentage of β cell labeling for Ki67 following adenoviral NFAT delivery to 5 or 6 different human islet preparations from 5 or 6 different donors (Figure 1, G–I). A negative control for adenoviral transduction, a Cre-expressing adenovirus, induced no human β cell proliferation, whereas the positive control, the DYRK1A inhibitor harmine in combination with the same Ad.Cre adenovirus, led to Ki67 immunolabeling in 2% of β cells, typical of results reported for DYRK1A small molecule inhibitors. In marked contrast, the 7 mouse or human NFAT adenoviruses — constitutively active or wild type — induced only very little or no Ki67 labeling, not approaching levels induced by harmine. The most effective were CA-mNFAT1 and CA-hNFAT3, but these averaged only 0.3% to 0.4% Ki67 labeling. Finally, we queried whether harmine might synergize with CA-hNFAT4 overexpression in β cells to drive higher rates of KI67 immunolabeling, but this did not occur (Supplemental Figure 1; supplemental material available online with this article; https://doi.org/10.1172/JCI157086DS1). Taken together, these findings make it clear that NFATs can be effectively overexpressed, and that the constitutively active versions effectively translocate to the nuclear compartment. Surprisingly, however, wild-type and CA-NFATs were unable to match the higher rates of proliferation observed in response to harmine or other DYRK1A inhibitors (7–17). These findings suggest that DYRK1A inhibitors may drive cell cycle entry and progression via additional pathways independent of NFAT signaling.

### The human insulinoma transcriptome predicts the DREAM complex as a central enforcer of human β cell quiescence.

We reasoned that an unbiased comparison of the transcriptomes of quiescent adult human β cells to those of the proliferating β cells in benign human insulinomas might provide a window into pathways important for driving β cell proliferation or enforcing quiescence. Accordingly, we turned to the weighted gene coexpression network analysis (WGCNA) we previously reported on our human insulinoma cohort in which we had identified 6 modules out of 52 that were significantly enriched in genes upregulated in insulinomas compared with FACS-isolated β cells (46, 47). Of the 6 modules, Bisque4 was of interest, as it was enriched for cell cycle control genes (Supplemental Figure 2A) (46). As the top 20 Bisque4 hub genes were G_2_/M genes exemplified by *CDK1*, *CENPF*, and *NUSAP* (Supplemental Figure 2B), we surmised that this module of genes reflected expression of β cells that had already entered G_2_M. To explore beyond the G_2_M process and find potential cell cycle–controlling genes acting upstream, we expanded the Bisque4 gene set to include genes that were co-correlated with the module eigenvector of Bisque4 (at *P <* 0.01). This resulted in a “Bisque4 module membership” group of 253 genes (Figure 2C, Supplemental Figure 3, and Supplemental Table 1) (46). We queried this geneset using iRegulon, a tool that searches for enriched transcriptional regulators underlying a coexpressed gene set using *cis*-regulatory sequence analysis (50). Among the top 6 enriched transcription factors identified of the Bisque4 module membership gene set, 4 factors (E2F4, TFDP1, MYBL2, and FOXM1) were noted as components of the DREAM and MMB complexes (Figure 2D, Supplemental Figure 2, and Supplemental Table 2 for full output). To more formally test this association with the DREAM complex, we next curated predicted direct target genes governed by TP53, DREAM, MMB-FOXM1, and RB-E2F as reported by Fischer et al. (39). We found that the Bisque4 module membership gene set was indeed significantly enriched in predicted targets of the DREAM (fold enrichment [FE] = 3.4-fold, *P =* 4.1 × 10^–15^), MMB-FOXM1 (FE = 7.8, *P =* 1.2 × 10^–19^), and RB-E2F complexes (FE = 3.3, *P =* 3.7 × 10^–8^), suggesting that these pathways may serve as the key gatekeepers for β cell quiescence and modulators of insulinoma proliferation (Supplemental Figure 4 and Supplemental Table 3). Collectively and sequentially, these observations predict that the repressive configuration of the DREAM complex maintains normal adult human β cells in a quiescent state, that interference with DYRK1A might induce proliferation in normal β cells, and that disruption of the DREAM pathway is an important contributor to proliferation in human insulinoma cells.

### Adult human β cells contain the repressive form of the DREAM complex.

**Figure 3:**
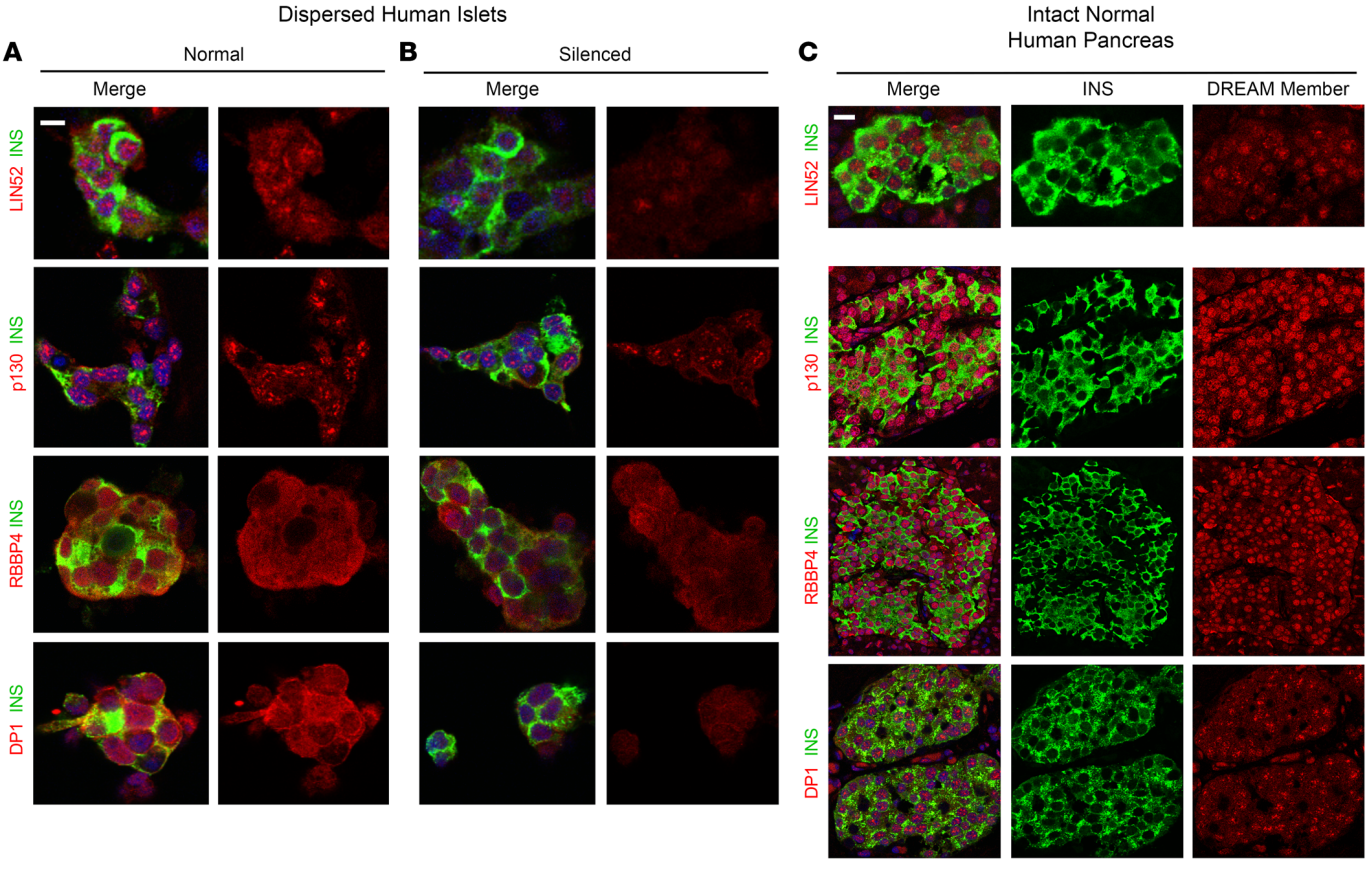
Immunohistochemical detection and subcellular localization of DREAM members in normal human pancreas. (**A**) Immunolabeling of LIN52, p130, RBBP4, and DP1 in dispersed human cadaveric islets. “Merge” indicates insulin (green) plus the DREAM member indicated (red). Note that all 4 DREAM members are present and are predominantly nuclear in human β cells. (**B**) Silencing in the same islets in the same experiments and islet donors following treatment with adenoviruses expressing shRNAs directed against the same 4 DREAM members, providing evidence of antibody specificity. (**C**) Immunolabeling of the same 4 DREAM members in normal human pancreas surgical samples, confirming expression of DREAM members and nuclear localization in the normal pancreas. See also Supplemental Figure 5, which shows immunoblots for E2F4, RBBP4, p130, and DP1, and immunolabeling for MYBL2. Antibodies used are described in Supplemental Methods. Each experiment shown is representative of 3 different human organ donor islets and 3 different human pancreas specimens. Scale bars: 10 μm.

To assess these possibilities, we explored 3 different human β cell transcriptome data sets for members of the DREAM complex within normal human β cells (Table 1) (46, 48, 49). We observed that adult human β cells reproducibly contain RNAs encoding all of the key members of the DREAM complex: LIN52, LIN54, RBBP4, p130, p107, E2F4, E2F5, DP1, along with lower levels of LIN9 and LIN37. Moreover, using immunohistochemistry, LIN52, RBBP4, p130, and DP1 were readily observed in the nuclei of dispersed human islets (Figure 3A) (immunohistochemistry-quality antisera for other MuvB members are not available). The specificity of antibody immunolabeling was confirmed by silencing the corresponding mRNA (Figure 3B). To confirm the presence of these DREAM proteins in normal β cells in situ, normal pancreas sections were explored, revealing strong nuclear immunolabeling for LIN52, p130, RBBP4, and DP1 (Figure 3C). Furthermore, E2F4, RBBP4, p130, and DP1 also were all observed in human islets by immunoblotting, and absent in human islets in which mRNAs encoding these proteins had been adenovirally silenced (Supplemental Figure 5A). Finally, MYBL2, a key driver of proliferation in the MMB complex (38–45), was undetectable at the mRNA (Table 1) or protein (Supplemental Figure 5B) level in quiescent adult human β cells. Thus, the canonical repressive DREAM members are present in quiescent human β cells. These results were supported by unbiased proteomic analysis of human islets (Table 1).

### Canonical DREAM target genes are silenced, but canonical G_1_/S genes are expressed, in human β cells.

Among the 1000 or more genes regulated by the DREAM complex are canonical G_2_/M genes involved in mitotic spindle formation, centriole separation, and other G_2_M processes, exemplified by *AURKA*, *AURKB*, *PLK1*, *CENPA*, *CENPF*, *FOXM1*, *BUB1*, *BIRC5*, *CDC25A*, *CDC25C*, *MELK*, and late-G_1_/S genes such as A- and E-cyclins (*CCNA*, *CCNE*) and *CDK1* (Figure 2B and Table 1) (38–45). RNA sequencing (RNA-seq) revealed that all of these are repressed in quiescent human β cells, along with MYBL2, also a direct target of DREAM repression (Figure 2B and Table 1). Thus, in addition to the presence of repressive DREAM members, most canonical DREAM targets are repressed in quiescent human β cells. Remarkably, this contrasts with canonical G_1_ members and their upstream drivers (Figure 2A), which are amply expressed in β cells, including CDK4, CDK6, all 3 D-cyclins, along with canonical cell cycle inhibitors (Table 1), also previously confirmed using immunohistochemistry (30–32). Thus, despite expression of G_1_ genes, adult human β cells are quiescent. In marked contrast to the G_1_ gene family, canonical DREAM target genes, as well as some G_1_/S target genes, are silenced in human β cells. Again, comparable results were observed using proteomic analysis (Table 1).

### DYRK1A inhibitor treatment and DYRK1A silencing activates DREAM target genes, but does not alter G_1_/S gene expression.

We next treated human islets for 72 hours with the DYRK1A inhibitor, harmine (10 μM), the maximally effective dose for inducing human β cell replication (Figure 1, G and H) (8–11, 15), and assessed DREAM target gene activation using RNA-seq of whole human islets. Table 2 illustrates that harmine treatment did not alter abundance of DREAM members (*LIN*s, *RBBP4*, *E2F4*, *p130*, etc). In contrast, harmine treatment did lead to increases in expression of essentially all of the canonical DREAM targets, including *MYBL2* (Table 2). Remarkably, these changes were associated with little or no change in expression of canonical G_1_/S genes encoding D-cyclins, CDK2, -4, -6, or the CDKI group (Table 2). Once again, comparable results were observed by proteomic analysis of whole islets (Table 2).

**Figure 4:**
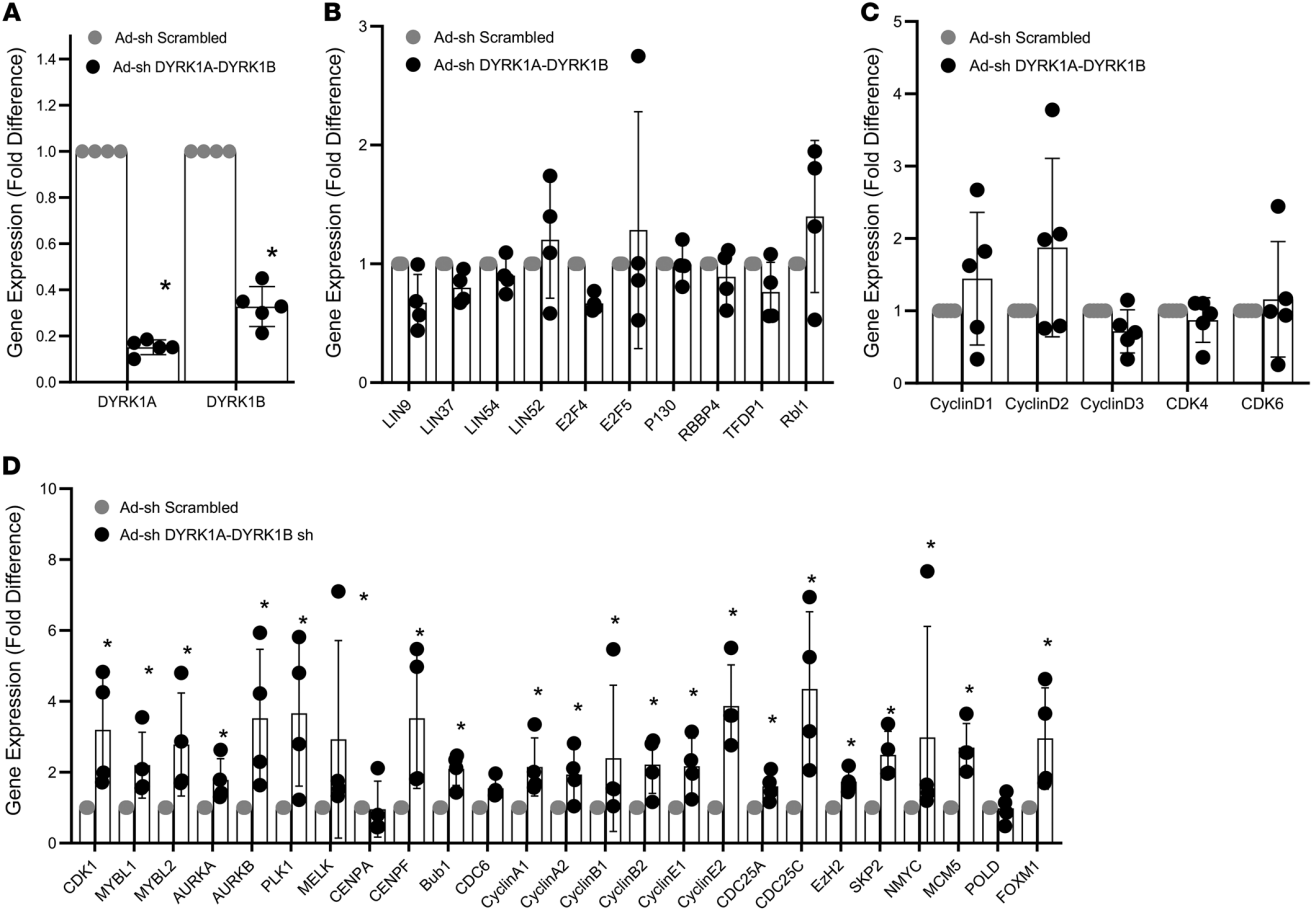
Silencing DYRK1A and DYRK1B induces expression of canonical DREAM target genes in human islets, but has no effect on G_1_/S pathway genes. All experiments are qPCR experiments on human islets transduced with a single adenovirus expressing shRNAs directed against both human DYRK1A and DYRK1B (see ref. 11 for details). The control is an identical adenovirus expressing a scrambled nonsense shRNA sequence. (**A**) Confirmation of silencing of DYRK1A and DYRK1B in human islets. (**B**) Absence of effect on DREAM family members by the same virus in the same experiment. (**C**) Absence of effect of the same virus in the same experiment on G_1_/S cyclins and CDKs. (**D**) Striking and uniform increases in DREAM target gene expression in human islets in response to silencing DYRK1A/B. *P < 0.05 , 2-tailed Student’s paired *t* test. *n =* 4–5 human islet donors for all experiments.

To independently ascertain whether these results were attributable to DYRK1A inhibition, we used a previously described adenovirus that expresses shRNAs that silence both DYRK1A and DYRK1B (11), since DYRK1B increases when DYRK1A is silenced in human islets, and since all DYRK1A inhibitors are also DYRK1B inhibitors (11). As shown in Figure 4, simultaneous silencing of DYRK1A and DYRK1B in adult human islets was effective in reducing DYRK1A and DYRK1B expression (Figure 4A), but had no effect on the abundance of DREAM members or canonical G_1_/S cyclins and CDKs (Figure 4, B and C). In marked contrast, silencing DYRK1A and DYRK1B resulted in very significant and almost universal increases in canonical DREAM target genes, notably including *MYBL1* and *MYBL2* (Figure 4D). Taken together, these findings support the notion that DYRK1A inhibitors induce human β cell proliferation by converting the DREAM/MMB complex from its repressive configuration into its proliferative configuration, while having little effect on canonical G_1_ members.

We further queried whether silencing other DREAM complex members might influence DREAM target gene expression by silencing E2F4 and E2F5, or all of the pRB family members (pRB, p107, and p130, encoded by *RB1*, *RBL1*, and *RBL2*, respectively) (Supplemental Figure 6). As with *DYRK1A/B* silencing, simultaneous silencing of E2F4 and E2F5 or all 3 pRB family members increased human β cell proliferation (Supplemental Figure 6A), but resulted in no alterations in expression of canonical DREAM members or G_1_/S cyclins (Supplemental Figure 6, B, C, E, and F). In contrast, each resulted in marked increases in expression of canonical DREAM target genes, including *MYBL1* and *MYBL2* (Supplemental Figure 6, D and G). Thus, the increases in DREAM target gene expression in response to harmine can be reproduced by, and directly attributed to, inhibition of DYRK1A, perhaps in combination with DYRK1B.

### Protein-protein interaction and ChIP studies confirm the operative existence of the repressive DREAM complex in adult human β cells.

**Figure 5:**
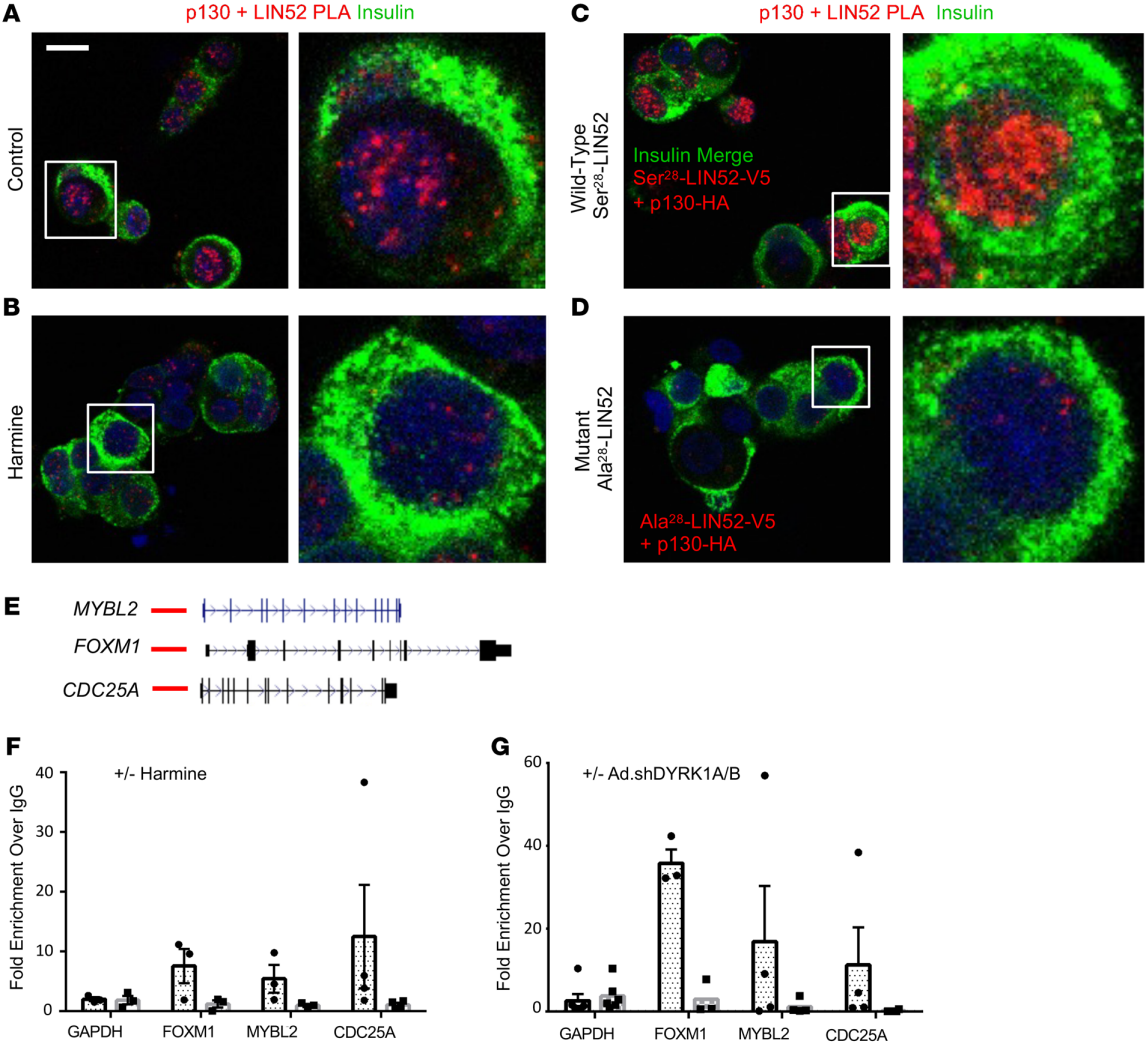
LIN52 and p130 colocalize with one another in human β cell nuclei, and assemble on DREAM target genes; DREAM complex disruption by harmine and genetic silencing of DYRK1A. (**A**) Proximity ligation assay (PLA) demonstrating colocalization of LIN52 and p130 in human β cell nuclei. The red nuclear signal indicates that the 2 proteins being examined are within <40 nm of one another. (**B**) Disruption of this interaction by DYRK1A inhibition using harmine. (**C**) Co-overexpression of wild-type LIN52 with a V5 epitope tag and wild-type p130 with an HA tag shows even stronger colocalization as compared with panel **A**. (**D**) Replacing Ser^28^ with Ala^28^ in LIN52 in otherwise identical constructs and experiments shown in panel **C** abolishes LIN52-p130 interactions. (**E**) UCSC Browser tracks for 3 canonical DREAM target genes, *MYBL2*, *FOXM1*, and *CDC25A*, with predicted upstream DREAM binding sites shown in red lines. (**F**) ChIP experiments showing interactions in normal islets between p130 and the 3 target genes in **E**, and disruption of these interactions by harmine. (**G**) Similar experiments to panel **F**, showing that silencing DYRK1A/B disrupts interactions of p130 with canonical DREAM target genes. Panels **A**–**D** are representative of experiments in 3 different human islet donors, and image intensity and statistics are shown in Supplemental Figure 9. Panels **F** and **G** include 3–4 donors as indicated. Data are presented as mean ± SEM. Scale bar: 10 μm.

While the studies described thus far suggest that the repressive form of the DREAM complex is active in quiescent human β cells, they do not formally prove its existence in functional terms. Accordingly, we next used proximity ligation assay approaches, which assess whether 2 proteins are within 40 nm or less of one another, to assess whether p130 and LIN52 might exist in physical association in adult human β cells. As shown in Figure 5A, LIN52 and p130 are in indeed in close proximity. However, upon harmine treatment of the same islets performed in separate chambers during the same experiment, the LIN52-p130 complex formation was reduced (Figure 5B). In separate experiments, 2 adenoviruses, one containing wild-type LIN52 with a V5 epitope tag and the other containing p130 with an HA tag, were overexpressed in human islets. This resulted in a substantial increase in intensity of the proximity ligation assay signal (Figure 5C). Finally, to explore the specific importance of Ser^28^ in LIN52, an adenovirus expressing a V5-tagged LIN52, in which Ala^28^ was substituted for Ser^28^, was cotransduced with the HA-tagged p130 adenovirus (Figure 5D). Despite an identical design and performance of the experiments in Figure 5, C and D, there was no evidence of interaction of Ala^28^-LIN52 protein with p130. Finally, as a negative control for the proximity ligation assay, we performed the same experiments in Figure 5, A and C, using only single antibodies, and observed no signal (Supplemental Figure 7). These results are quantified in Supplemental Figure 8. These observations provide strong support for the notion that p130 and LIN52 physically associate within the nuclei of quiescent adult human β cells, that this association requires phosphorylation of the DYRK1A target, Ser^28^ in LIN52, and can be disrupted by genetically or pharmacologically interfering with DYRK1A.

The repressive form of the DREAM complex binds to consensus DP1, E2F4, p130 binding sites (CDEs) of DREAM target genes (38–45). Thus, we next sought to determine whether DREAM repressive members were in physical contact with CDE regulatory sequences of canonical DREAM target genes in quiescent human β cells. As target genes, we selected 3 canonical DREAM targets, *MYBL2*, *FOXM1*, and *CDC25A* (Figure 5E), and targeted p130 for immunoprecipitation. As can be seen in the ChIP analysis in Figure 5, F and G, p130 does indeed interact with regulatory regions of *FOXM1*, *MYBL2*, and *CDC25A*. More importantly, treatment with the DYRK1A inhibitor, harmine, or silencing DYRK1A and DYRK1B, leads to disruption of these interactions. Taken together, these studies demonstrate that in addition to the canonical DREAM interactions between p130 and LIN52 in the nucleus of quiescent adult β cells (Figure 5, A–D), p130 physically associates with regulatory regions of canonical DREAM target genes (Figure 5, E–G), and this repressive configuration can be disrupted by pharmacologic and genetic DYRK1A interference.

### The DREAM complex is present in human α cells.

Reasoning that quiescence in other islet endocrine cells may reflect the presence of the DREAM complex, we also assessed the presence of DREAM members in α cells in normal intact human pancreas. As observed in Supplemental Figure 9, A–D, DREAM members LIN52, DP1, RBBP4, and p130 were also present in nuclei of α cells. Quantification of these 4 factors in human α cells and in human β cells revealed that these DREAM members are present in the nuclei of almost 100% of α and β cells (Supplemental Figure 9, E and F). Finally, DREAM members p130 and LIN52 appear to be in direct association as assessed using proximity ligation assay (Supplemental Figure 10, A–H), as was observed in human β cells.

## Discussion

Here we report 4 fundamental advances in our understanding of mammalian cell cycle control. First, we provide the first example to our knowledge of the contribution of the DREAM complex in maintaining quiescence in a normal mammalian cell type. Second, we demonstrate the presence and the central role of the repressive configuration of the DREAM complex in maintaining quiescence in the adult human β cell, a cell type notorious for its resistance to proliferation, as well as the normal human α cell. Third, we demonstrate that the repressive DREAM complex configuration can be converted to its pro-proliferative MMB counterpart by DYRK1A inhibition, thereby moving adult human β cells from quiescence to active replication. Fourth, we provide a revised model explaining how human β cell regenerative drugs — the DYRK1A inhibitors — are able to coax previously quiescent human β cells to reenter the cell cycle. These observations have important implications for diabetes therapy, and also for cancer and developmental biology.

The mammalian DREAM complex has been studied principally in continuously growing cell lines derived from common cancers, exemplified by breast, lung, prostate, esophageal, and ovarian cancers and certain leukemias, in which the repressive form of DREAM has been disrupted, and/or in which misexpression of MYBL1 or MYBL2 results in them acting as oncogenes (38–45). As a result, in these scenarios, MMB members serve as oncogenic drivers. In contrast, we report here, to the best of our knowledge for the first time in any normal human cell type, that the repressive form of the DREAM complex plays a central role in enforcing or maintaining quiescence in a mature, healthy, normal cells.

In the context of diabetes, it has long been clear that adult human β cells are quiescent, terminally differentiated, and resistant to attempts to induce regeneration or proliferation. This replicative refractoriness has been attributed variously to multiple processes, including chromatin configurations and/or DNA methylation patterns that enforce quiescence through repression of D-cyclin and CDK genes (7, 33–35, 46, 47, 51); activation of cell cycle inhibitors, notably p16^INK4A^ encoded by *CDKN2A* and p57^KIP2^ encoded by *CDKN1C* (7, 36, 52, 53); exclusion of cell cycle molecules from the nucleus, and restriction to the cytoplasm (31, 32); expression of long noncoding RNAs or microRNAs; and/or, inactivation or failure of upstream mitogenic signaling pathways, for example RASSF1 signaling (54). Whether and in what manner these processes may interact with DREAM-complex biology will be an important avenue to pursue in future studies.

Several groups have reported that small molecule inhibitors of DYRK1A are effective and reproducible inducers of adult human β cell proliferation (7–17). Although most DYRK1A inhibitors also interfere with other mammalian kinases, it is clear that DYRK1A is the relevant antimitogenic target, since parallel and comparable degrees of human β cell proliferation can be achieved by inhibiting DYRK1A expression in human β cells (7–11, 15); conversely, overexpression of DYRK1A in adult human islets blocks β cell proliferation in response to small molecule DYRK1A inhibitors such as harmine, INDY, and 5-IT (7–11). We have also observed that silencing DYRK1B has no effect on human β cell proliferation (11). However, when DYRK1A is silenced in human β cells, there is a compensatory increase in DYRK1B expression (11). As a result, simultaneous silencing of DYRK1A and DYRK1B, compared with silencing DYRK1A alone, affords greater increases in human β cell proliferation (11). Since all DYRK1A inhibitors studied are also comparably effective DYRK1B inhibitors (7–17), we elected to silence both DYRK1A and DYRK1B (Figure 4). Whether there is a specific role of DYRK1B in DREAM-complex biology is unexplored to our knowledge.

The most widely held mechanistic paradigm used to explain how DYRK1A enforces quiescence in human β cells is summarized in Figure 1A, and indicates that activation of proliferation results from 4 sequential steps (7–26): (i) activation of calcineurin leading to dephosphorylation of NFATs, sequestered in the cytoplasm by 14-3-3 scaffold proteins; (ii) trafficking of dephosphorylated NFATs to the nucleus; (iii) NFAT binding to, and transactivation of, regulatory regions of genes encoding cyclins and CDKs, and repression of genes encoding cell cycle inhibitors; resulting in, (iv) initiation of cell cycle entry at G_1_/S (23, 24). In this broad paradigm, nuclear DYRK1A rephosphorylates NFATs, forcing their expulsion from the nucleus, thereby interrupting the mitogenic cascade, and forcing cells to return to quiescence. Thus, by regulating NFAT trafficking and function, DYRK1A serves a “brake” on proliferation; conversely, DYRK1A inhibitors remove this brake, permitting β cells to enter the cell cycle.

We and others have provided data to support steps i and ii (7–26), but had not rigorously assessed the final 2 steps by asking whether nuclear transit of NFATs would actually drive human β cells to enter the cell cycle. We had wondered whether the NFAT scenario fully explained DYRK1A inhibitor mechanisms of action for 2 reasons. First, although we showed that interference of calcineurin-NFAT interactions using the short peptide inhibitor, VIVIT, or the calcineurin inhibitor, FK506 (Figure 1A), reduced human β cell proliferation, neither compound completely blocked harmine-induced proliferation (8), suggesting that additional, calcineurin- and NFAT-independent pathways may be important. Second, review of prior reports provided little direct evidence of induction of adult human β cell proliferation by NFAT family members (23, 27, 28). Thus, to more fully explore an NFAT contribution to the model, we elected to overexpress human NFATs in human β cells and assess proliferation. To our surprise, although harmine induced ample proliferation, assessed by Ki67 immunolabeling, wild-type NFATs induced only modest proliferation (Figure 1, G–I). We then turned to 2 constitutively active mouse NFAT constructs previously reported to induce human β cell proliferation (27, 28), but again observed only modest proliferation (Figure 1, E and G–I). We then used 3 constitutively active nonphosphorylatable human NFATs (containing 21 and 8 serine-to-alanine substitutions) (18–21), and observed that while they did enter the nucleus, they also produced little or no proliferation. These events suggest that DYRK1A inhibitors might interact with additional, previously unrecognized pathways able to activate β cell proliferation.

Searching for such unrecognized pathways in human β cells, we reexplored our differentially expressed gene sets derived from RNA-seq from quiescent human β cells as compared with human insulinomas, which contain proliferating human β cells (46). Further analysis of these data sets (Figure 2 and Supplemental Figures 2–4) pointed to the DREAM complex as a likely repressor of proliferation in human β cells, and to its disruption within proliferating β cells in insulinomas. Against this background, DYRK1A is well known to phosphorylate Ser^28^ in LIN52, an event that converts the proliferative MMB configuration to the repressive DREAM complex, thereby inducing cell cycle arrest or quiescence in many cancer cell types (38–45). Seeking evidence for DREAM complex presence and activity in human β cells, we searched β cell RNA-seq data sets from ourselves and others (46, 48, 49), and observed that repressive DREAM members are reproducibly present in β cells, and that their canonical targets are repressed, while canonical G_1_/S members are relatively abundant (Table 1). We confirmed the presence of key DREAM member proteins in human β cells, both in culture and in normal intact human pancreas specimens (Figure 3 and Supplemental Figure 5). We also observed that harmine treatment or DYRK1A/B genetic silencing increased the expression of canonical DREAM target genes, including the central target, *MYBL2* (Table 2 and Figure 4), but had little effect on expression of canonical G_1_ gene targets (Table 2 and Figure 4). One of the defining features of the repressive form of the DREAM complex is physical association of LIN52 and p130 (38–45), a finding we demonstrate in quiescent β cells, and which is reversed by treatment with harmine (Figure 5, A–D, and Supplemental Figure 7). Importantly, mutating the DYRK1A target, Ser^28^ in LIN52 to Ala^28^, abolished the LIN52-p130 interaction (Figure 5, C and D). Finally, we observed that p130 bound to regulatory regions of 3 canonical DREAM target genes, *FOXM1*, *MYBL2*, and *CDC25A*, in quiescent human islets, and this interaction was disrupted by inhibiting DYRK1A, both pharmacologically with harmine or genetically by silencing DYRK1A/B (Figure 5, E–G). These observations make it unequivocally clear that the DREAM complex represses proliferation and enforces quiescence in human β cells, and illustrates how DYRK1A inhibitors are able to activate their proliferation. Remarkably, we also find evidence of the repressive form of the DREAM complex in human α cells as well (Supplemental Figures 9 and 10).

In retrospect, there have been clues to suggest that the DREAM complex may be relevant to control of human β cell replication. For example, Abdolazimi et al. have suggested that recombinant LIN52 is a direct phosphorylation target of recombinant DYRK1A, and that DYRK1A inhibitors increase expression of *MYBL2* (16); Gannon et al. and Davis et al. have shown that FOXM1 contributes to β cell proliferation in mouse models of pregnancy and other pro-proliferative maneuvers (55–57); El Ouamaari et al. and Dai et al. have shown that canonical DREAM targets CENP, PLK1, FOXM1, CDK1, and A-cyclin, but not canonical G_1_/S cyclins or CDKs, increase in response to mitogenic stimuli in β cells (26, 58); and Klochendler et al. have shown that mouse β cells induced to replicate by constitutively active glucose analogs induce a repertoire of G_2_M genes (59), which in retrospect are canonical targets of the DREAM complex. Indeed, our own studies with harmine alone or in combination with GLP1 receptor agonists or with TGF-β superfamily inhibitors showed a recurrent pattern of activation of DREAM target genes, without changes in canonical G_1_/S genes (8–10), reflecting, in retrospect, conversion of the repressive DREAM complex in human β cells to the pro-proliferative MMB configuration.

These findings require reassessment and reinterpretation of prior reports that suggested NFATs as the primary drivers of β cell proliferation. This presumption likely derives from studies in T cells in which NFATs do appear to drive proliferation in association with nuclear translocation (18–21). Studies by Crabtree et al., Mognol et al., and Rao et al. also showed that substituting multiple serines for alanines in serine-rich regions of NFATs allowed them to transit to the nucleus and transactivate target genes in T cells (18–21). Goodyer et al. and Demozay et al. suggested a similar mechanism, by showing that mouse NFATs bind to cell cycle genes in mouse β cells by ChIP analysis (23, 25), and Dai et al. suggest that NFATs may be important in mediating proliferation in juvenile, but not adult, human β cells (26). Simonett et al. have suggested that constitutively active mouse NFAT1 and -2 can drive mouse and human β cell proliferation, as assessed primarily by cellular uptake of ^3^H-thymidine, an assessment that did not specifically identify proliferation in β cells, as pointed out by the authors (27, 28). Abdolazimi et al. have also noted that inhibition of the calcineurin/NFAT pathway only partially attenuated β cell proliferation induced by DYRK1A inhibitors (16). Collectively, the current findings suggest an alternative to the conventional NFAT model (Figure 1A); DYRK1A inhibitors act to drive human β cell proliferation principally via disruption of the repressive DREAM complex.

These findings do not exclude an important role for NFATs in human β cell biology. In our own studies, we and others have shown that NFATs do indeed translocate to the nucleus in response to DYRK1A inhibitors (8, 13, 15, 16), and also appear to play a central role in insulin gene expression in human insulinomas (46, 47). Goodyer et al. have made a compelling case for a direct transcriptional role for NFATs in controlling expression of genes involved in β cell differentiation and neurosecretory function (23). Demozay et al. have shown that NFATs translocate to the nucleus in response to glucose stimulation, and transcriptionally and directly activate *IRS2* gene expression (25). NFATs are also known to partner with AP-1 factors, SMADs, and STATs to coregulate key T cell genes (21). Most recently, Simonett et al. have shown, using ChIP-seq, that NFAT binding sites are abundant in human islets, that NFATs colocalize with, and coimmunoprecipitate with, FOXP family transcription factors in human islets, and that NFAT-FOXP dimers coregulate target genes (28). Finally, Dai et al. have shown that NFATs are involved in driving proliferation in human β cells derived from children, but not adults (26). Thus, future studies will undoubtedly elucidate additional important roles, mechanisms, and functions for NFATs in human β cell biology.

It is clear that conventional G_0_-G_1_ molecules in Figure 2A must operate cooperatively and sequentially with DREAM and its downstream targets in Figure 2B. In normal cell cycle progression paradigms, DREAM-MMB conversion is envisioned as being activated during, and as a consequence of, successful G_1_/S entry; both pathways are essential for orderly cell cycle progression. Nonetheless, as observed in Figure 4 and Table 2, there is little apparent effect of DYRK1A inhibition on canonical G_1_/S pathway molecules. These events suggest that DREAM and therefore G_2_/M pathways are the principal targets of DYRK1A inhibition.

**Figure 6:**
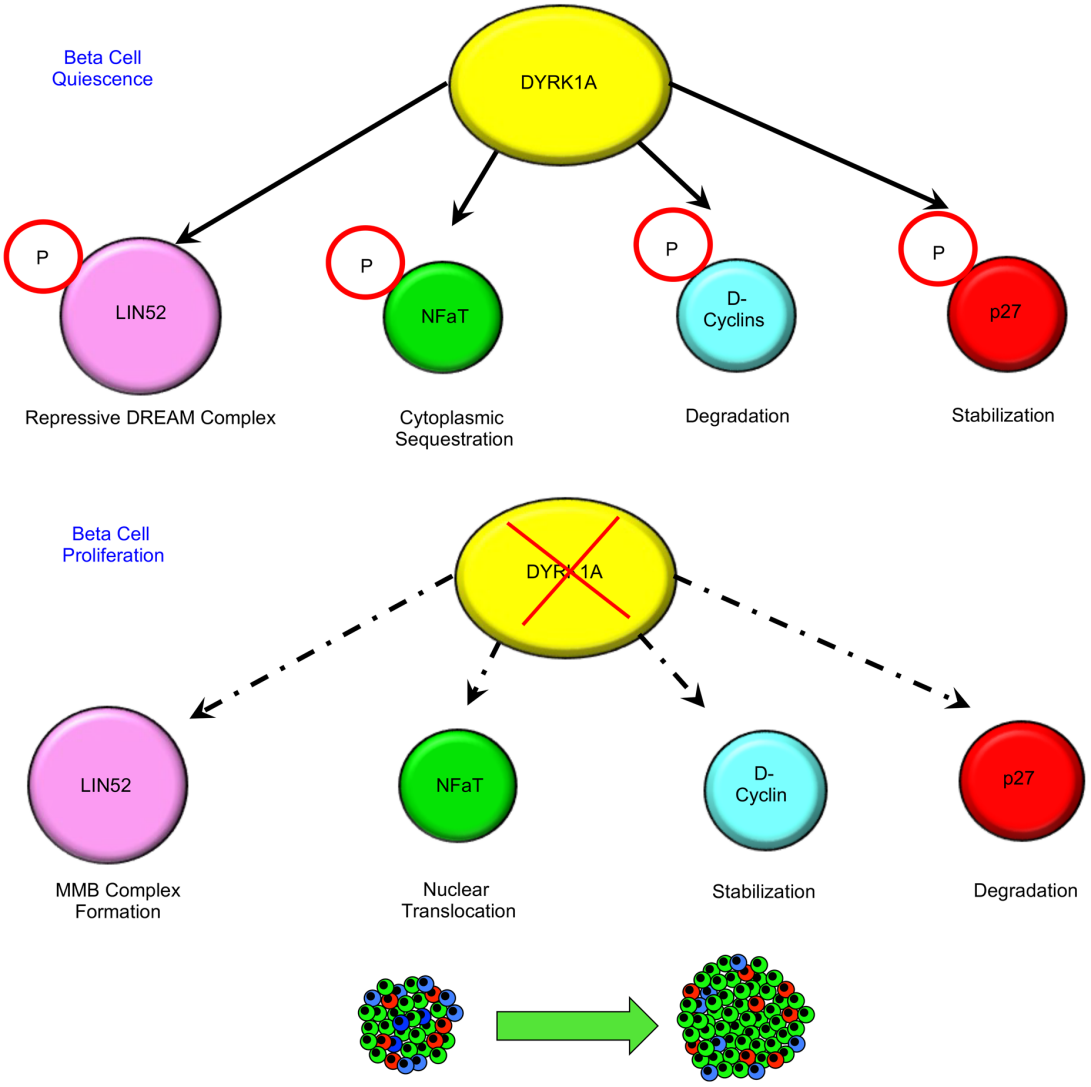
A model illustrating well-documented and potential human cell cycle pathways and targets through which the DREAM complex enforces quiescence, and the mechanism(s) through which DYRK1A inhibition leads to β cell proliferation. The size of the circles indicates the strength of evidence for each pathway in human β cells. “P” surrounded by a circle in the top rows indicates that a target protein is phosphorylated by DYRK1A. See main text for additional details.

In addition, it is also likely that DYRK1A inhibition may contribute to cell cycle progression through engagement of additional cellular pathways. For example, Annes et al. and others have shown that recombinant DYRK1A phosphorylates and thereby stabilizes the cell cycle inhibitor p27^CIP2^, an event that would favor quiescence (16). Also, several authors have indicated that DYRK1A can phosphorylate, and thereby destabilize D-cyclins (60, 61), events that also would favor cell cycle arrest. Figure 6 summarizes these multiple potential pathways to proliferation mediated by DYRK1A inhibition. We suggest that all of these are potentially important, but that the DREAM arm of these models currently has the strongest experimental support in human β cells.

Finally, these studies make one additional important point; they provide a fourth example validating the concept that human insulinomas can serve as a data mine for revealing pathways that can be manipulated to induce human β cells to replicate. More specifically, prior work from this data set has suggested that DYRK1A inhibitors such as harmine (7–17), TGF-β superfamily inhibitors such as LY364947 (9, 46), GLP1 receptor agonists that activate pathways downstream cAMP/PKA signaling such as CREB, CREBBP, and p300 (10, 46), and now DREAM complex (here) may be valuable drug targets for human β cell regeneration.

To conclude, these studies provide a current working model to clarify the mechanisms of action whereby DYRK1A inhibitors induce human β cell proliferation (Figure 2B and Figure 6). They also raise additional unresolved questions. For example, does DREAM-mediated repression of the cell cycle operate independently from, or cooperatively with, epigenetic mechanisms, for example, by recruiting, or being recruited by, Polycomb or Trithorax members to regions of closed or inaccessible chromatin? And, how do the histone acetylases and deacetylases highlighted by the iRegulon DREAM complex findings in Figure 2D participate in DREAM/MMB biology in general and in β cells in particular? And how are G_1_/S events, which must precede G_2_/M events, coordinated and synchronized following DYRK1A inhibition? And in addition to β and α cells, does DREAM play a role in δ, pancreatic polypeptide (PP), and other islet endocrine cells? These questions and others will provide ample opportunities for future studies.

## Methods

### Human islets and pancreatic sections.

Isolated deidentified human pancreatic islets from otherwise normal organ donors were provided by the NIH Integrated Islet Distribution Program (IIDP, https://iidp.coh.org), Prodo Laboratories, The Alberta Diabetes Institute, and the Transplant Surgery Department, University of Chicago. Details and demographics of the 53 donors and islet preparations are provided in Supplemental Table 4. Donor ages ranged from 19 to 68 years old. The mean age (±SEM) was 43.3 ± 12.8 years; mean BMI (±SEM) was 27.1 ± 5.5 (range 10–47.6); 42 of 53 were male; 25 were White, 15 Hispanic, 4 Asian, 5 Black, and 4 were not identified; mean cold ischemia time 481.4 ± 242 minutes (range 213–1080 minutes); and islet purity ranged from 75% to 95% (mean 87.5% ± 4.6%).

### Detailed methods.

Complete methods are provided in the Supplemental Methods section.

### Statistics.

Statistical analyses were performed using 2-tailed Student’s paired *t* test as described in the figure legends. *P* values less than 0.05 were considered to be significant. Detailed bioinformatics statistics are provided in the Results section, *The human insulinoma transcriptome predicts the DREAM complex as a central enforcer of human β cell quiescence*.

### Human study approval.

Human islets were purchased from the islet isolation centers listed above and in Supplemental Table 4 in accord with NIH and Icahn School of Medicine Human Islet Policy. Deidentified human pancreas histologic sections were provided by the Mount Sinai Biorepository and Pathology Core. Written informed consent for research and IRB approval was obtained by the providing institution or department.

## Author contributions

PW, EK, CA, HW, MB, HGR, XL, OW, HL, and LC performed experiments. PW, EK, CA, HW, JAP, DKS, LL, and AFS analyzed data. RIB provided human pathology samples. PW, CA, EB, DH, JAD, and AFS conceived of the studies. PW, EK, JAD, and AFS wrote the manuscript.

## Supplementary Material

Supplemental data

Supplemental tables 1-4

## Figures and Tables

**Table 2:**
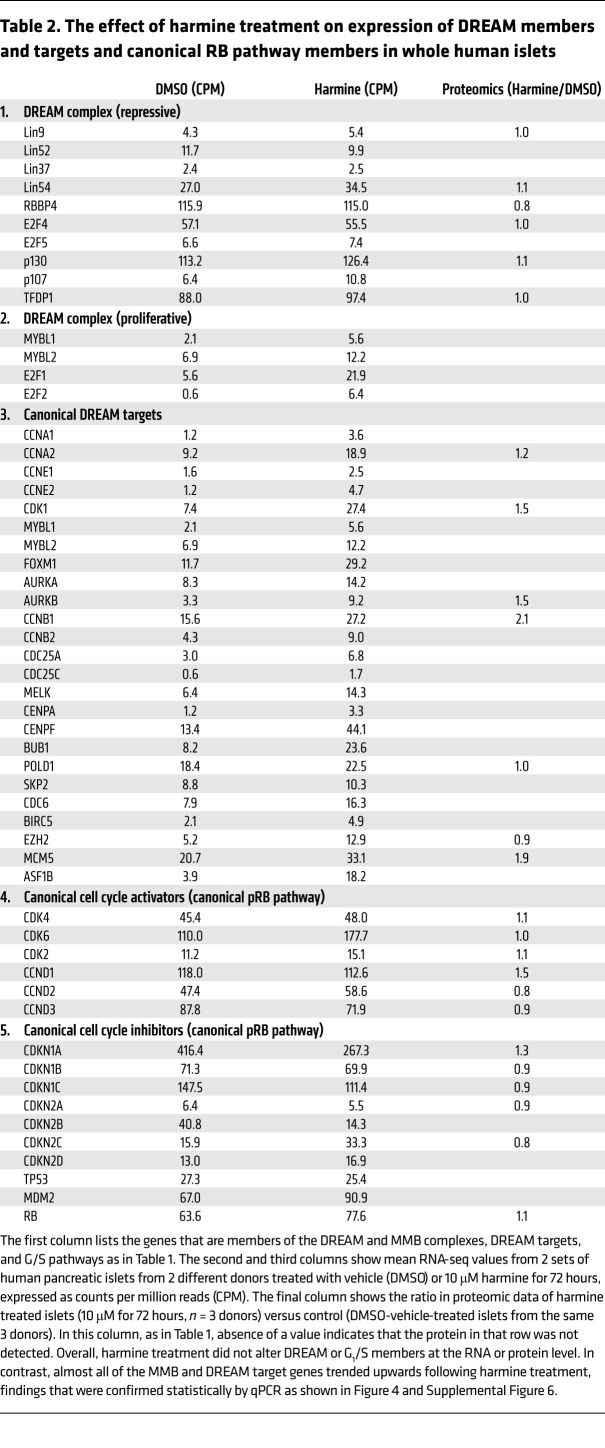
The effect of harmine treatment on expression of DREAM members and targets and canonical RB pathway members in whole human islets

**Table 1:**
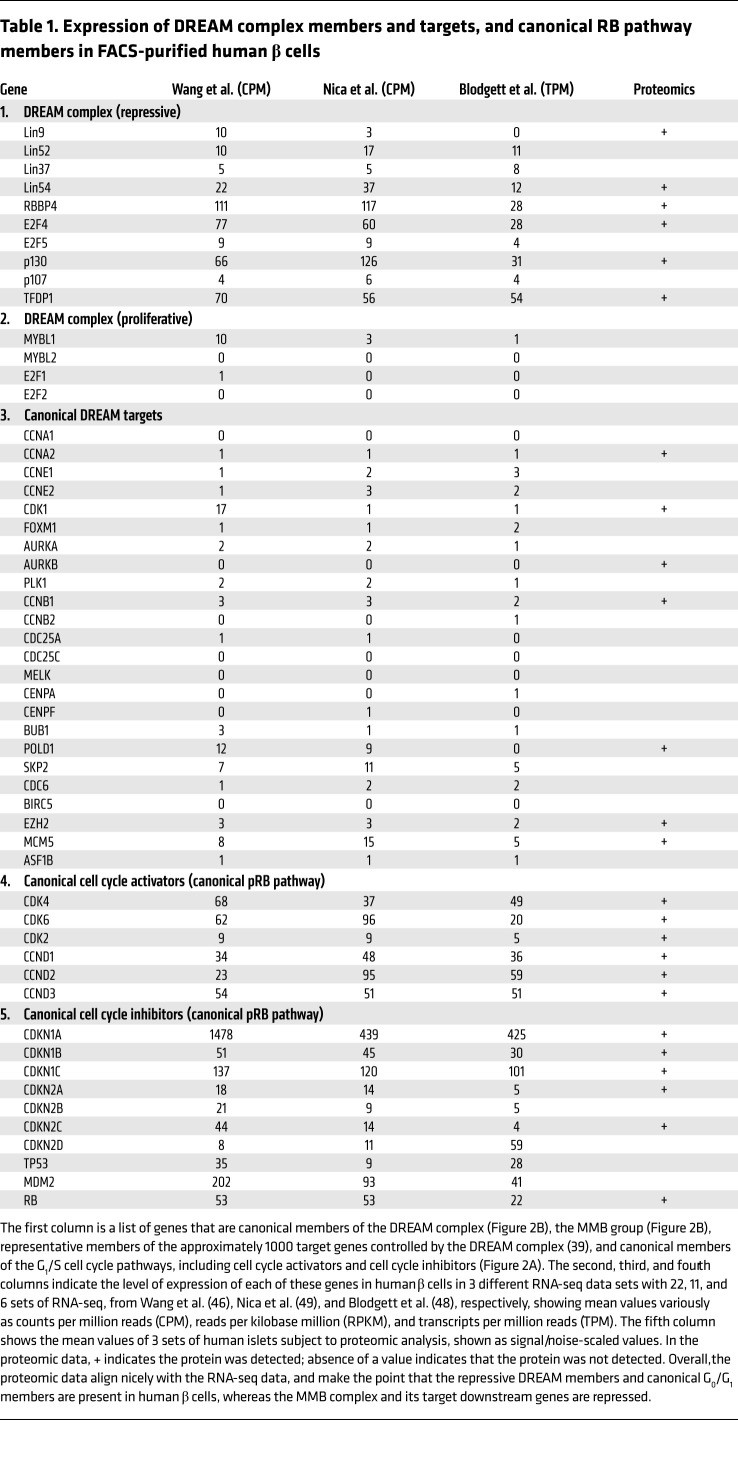
Expression of DREAM complex members and targets, and canonical RB pathway members in FACS-purified human β cells

## Version 1 06/14/2022

In-Press Preview

## Version 2 08/01/2022

Electronic publication
