# Supplementary material for: Disrupting the DREAM complex enables proliferation of adult human pancreatic β cells
Source: J Clin Invest. 2022 Aug 1;132(15):e157086. doi: 10.1172/JCI157086 (PMC9337832; doi:10.1172/JCI157086)
Supplement: Supplemental data [file jci-132-157086-s115.pdf]

Supplemental Methods, Figures and Tables

Disrupting the DREAM Complex
Enables Proliferation of Adult Human Pancreatic Beta Cells

[1,2,#]Peng Wang, [1,2,#]Esra Karakose, [3#]Carmen Argmann, [10]Huan Wang, [2]Metodi Balev, [4]Rachel Brody, [11,12]Hembly Rivas, [11]Xinyue Liu, [1,2]Olivia Wood, [1,2]Hongtao Liu, [1,5]Lauryn Choleva, [6,7,8]Dan Hasson, [6,7,9]Emily Bernstein, [11]Joao A. Paulo, [1,2]Donald K. Scott, [1,2]Luca Lambertini, [11,12,#]James A. DeCaprio, [1,2,#]Andrew F. Stewart.

From the [1]Diabetes Obesity Metabolism Institute, [2]Department of Medicine, [3]Department of Genetics and Genomic Sciences, [4]Department of Pathology, [5]Department of Pediatrics, [6]Department of Oncologic Sciences, [7]The Tisch Cancer Institute, [8]Bioinformatics for Next Generation Sequencing (BiNGS) Shared Resource Facility,[9]The Graduate School of Biomedical Sciences, Icahn School of Medicine at Mount Sinai, New York, NY 10029; [10]Sema4, Stamford CT 06902; [11]Dana Farber Cancer Institute, Boston MA 02215, and [12]Department of Medicine, Brigham and Women's Hospital, Harvard Medical School, Boston MA 02115.

Supplemental Detailed Methods.

Supplemental Figure 1. Effect of harmine on nuclear abundance of hNFAT4 and beta cell proliferation.

Supplemental Figure 2. Weighted Gene Co-Expression Analysis (WGCNA) of human beta cells vs. human insulinomas.

Supplemental Figure 3. Enlarged version of Fig. 2C.

Supplemental Figure 4. Network of predicted targets governed by TP53, DREAM, MMB-FOXM1 and RB-E2F that are also genes in the insulinoma Bisque4-Module Membership geneset.

Supplemental Figure 5. Confirmation of canonical DREAM members in human islets in by immunoblot.

Supplemental Figure 6. Silencing E2F4 + E2F5 or all three Rb family augments DREAM target gene expression but not the DREAM members or the CDK4/6-Cyclin D family.

Supplemental Figure 7. A negative control for the Proximity Ligation Assay.

Supplemental Figure 8. Intensity of PLA fluorescence in nuclei of human insulin-positive beta cells

Supplemental Figure 9. Intensity of PLA fluorescence in nuclei of human insulin-positive beta cells.

Supplemental Figure 10. Proximity assay reveals interactions of DREAM members in human alpha cells, and their disruption by harmine or LIN52 Ser^{28} Mutation.

Supplemental Table 1. The members of the Bisque4-MMP <0.01 geneset: 253 genes, related to Figure 2. (.xls Supplemental Table).

Supplemental Table 2. Full output of iRegulon analysis of the Bisque4-MMP geneset using the 500bp promoter regions (.xls Supplemental Table).

Supplemental Table 3. Results of Enrichment Analysis of Bisque4-MMP geneset (253 genes) in Dream Target-Associated Genesets Curated from Fischer et al (39) (Related To Suppl. Fig. 3) (.xls Supplemental Table).

Supplemental Table 4. Description of Human Pancreatic Islets Used (.xls Supplemental Table).

Supplemental Table 5. Primers Used For qPCR Experiments.

Supplemental Methods

Human Islet Dispersion and virus infection. Islets were centrifuged at 1500 rpm for 5 min, washed twice in phosphate-buffered saline (PBS), re-suspended in 1 ml of Accutase, and incubated for 10 min at 37°C. During digestion, the islets were dispersed by gentle pipetting up and down every 5 min for 10 sec. Complete RPMI medium containing 11 mmol/L glucose, 1% penicillin/streptomycin with 10% fetal bovine serum (FBS) was then added to stop the digestion. Dispersed cells were then centrifuged for 5 min at 1500rpm, the supernatants removed, the pellets re-suspended in complete medium, and cells then plated on coverslips with 30 µl cell suspension per coverslip. Poly-D-Lysine/Laminin-treated cover slides or chamber slides were used. Cells were allowed to attach for 2 hr at 37°C or were transduced with adenovirus for 2 hr. After 2 hr, 500 µl complete RPMI medium was added in each well to terminate the adenoviral transduction. Cells were cultured for 24–96 hr as described in the Figure legends. Detailed protocols are provided in reference 62.

Chemicals: Harmine HCl was purchased from Sigma, Cat # 286044.

Compound Treatments. For compound treatments, dispersed islet cells were allowed to recover on coverslips for 24 hr. Complete medium was then replaced with medium containing harmine or vehicle (0.1% DMSO) for 72-96 hr, at the doses described in the Figures. For the combination treatment with virus and compounds, islet cells were transduced first, then treated cells with compound 24 hours later.

Adenoviruses and Transduction. Adenoviruses for overexpression were prepared as described previously (8,11). Unless otherwise described, all transductions were performed using 150 moi (based on cell number) for two hours, and studies performed 72 hours later. Adenoviruses encoding human HA-p130 or V5-Lin52 were prepared as described (8,11) using cDNAs encoding p130 and Lin52 obtained from Harvard PlasmID Database (https://plasmid.med.harvard.edu/). The HA sequence was added to N-terminal of p130 by PCR; the V5 sequence was added to the Lin52 N-terminal by PCR. Corresponding primers are shown as follows: HA-p130 Forward: TACTAC GGTACC ACCATG TAC CCA TAC GAT GTT CCA GAT TAC GCT; p130 Reverse: TACTAC GATATC TCA GTGG GAAC CACG GTCA TTAG. V5-Lin52 Forward: TACTAC GGTACC ACCATG GGT AAG CCT ATC CCT AAC CCT CTC CTC GGT CTC GATTCTACGATGGCGTCTCCCACAGACGGG; V5-Lin52 Reverse: TACTACCTCGAG CTACTTCTTGGGCTTCTCTAGAAT. Mutant V5-LIN-52 was generated by using site-directed mutagenesis (Q5 E0554S Kit, New England Bio) to change Ser28 to Ala28. Ad.*NFATC1* is described in reference 8. Ad mouse ca-nfatc1 and ca-nfatc2 were gifts from Drs. Alan Attie and Mark Keller, Department of Biochemistry, University of Wisconsin. NFAT4 (NFATC3), CA-NFAT2 (CA-NFATC1), CA-NFAT3 (CA-NFATC4) cDNAs were obtained from Addgene (https://www.addgene.org). Ad human CA-NFAT4 (CA-NFATC3) was generated by site-directed mutagenesis as above, altering S163A, S165A, S168A, S169A, A177A, S180A, S181A, S184A, S186A, S207A, S211A, S215A, S236A, S240A, S244A, S247A, S248A, S277A, S288A, S292A, and S304A. cDNAs were cloned and inserted into PENTR2B vector, then recombined with adenovirus backbone plasmid and packaged into 293A cells, to generate the respective adenoviruses.

For adenoviral silencing, four target shRNA sequences for each target gene were designed using the ThermoFisher RNAidesigner online tool. Target sequences were cloned into block-iT U6 RNAi entry vector (K494500, Invitrogen), and silencing efficiency was evaluated in 293 cells. The most effective target sequences were used to generate Ad-shRNAs in the Block-iT adenoviral RNAi vector (K494100 Invitrogen). Adenoviruses for silencing RBL2/p130, RBL1/P107, Rb1, Lin52, E2F4, E2F5, TFDP1 and RBBP4 were prepared using U6 RNAi entry vector (45-0511, Invitrogen). To silence multiple genes in one virus, we cloned the U6-shRNA sequence by PCR and ligated this into modified PENTR 2B gateway vector as described in detail previously (11). The virus that silences both DYRK1A and DYRK1B simultaneously has been reported previously (11). The same method was used to generate adenoviruses able to silence both E2F4-E2F5, or pRb, p107 and p130 using a single virus. For shRNA target sequences, Ad.shDYRK1A against DYRK1A has been reported previously (8), for DYRK1B: GCTGGAGCGCTACGAAATTGA; p130 GCCCTGTACTGTGTCTGAAAT; LIN52: GGAGTCTCACCACGGCTAATT; E2F4: GCGGCGGATTTACGACATTAC; TFDP1: GCGCGTCTACGATGCCTTAAA: RBBP4: GGTCATACTGCCAAGATATCT; P107: GCACAAGAGGTACATTCAACT; RB1: GGAAAGGACATGTGAACTTAT; and, E2F5: GGCAGATGACTACAACTTTAA.

Immunocytochemistry and Antisera. Immunocytochemistry was performed on 4% paraformaldehyde-fixed (15 min), Accutase-dispersed human islets plated on coverslips as described (8). For human pancreatic tissue immunolabeling, citrate pH 6.0 buffer and low pressure cooker treatment for 20 min was used for antigen retrieval. Primary antisera were: Ki67 (RM-9106-s1, Thermo Scientific); C-peptide (DSHB GN-ID4); p130 (Life Technologies MA5-14305); Glucagon (Abcam, ab10988 ab108426); LIN52 (Life technologies PA5 81210); E2F4 (Life technologies Ma5-11276); MYBL2 (EMD Millipore MABE886); TFDP1 (Life Technologies PA5-102770); RBBP4 (Life technologies, Ma1-23273); V5 (Life Technologies); HA (Santa Cruz Sc-805); NFAT1 (Abcam ab2722); NFAT2 (BD Pharmingen 556602); NFAT3 (Santa Cruz SC-13036); NFAT4 (Santa Cruz SC-8321); and, alexa fluor-conjugated anti-glucagon (R&D Systems IC 1249C).

Immunoblotting Procedures. Immunoblots were performed on whole human islets as described in detail previously (8). Primary antisera were E2F4 (Life technologies Ma5-11276); p130 (BD Pharmingen 610261); TFDP1 (Life Technologies PA5-102770); RBBP4 (Abcam Ab1765); and, GAPDH (Sc-25778, Santa Cruz).

Proximity Ligation Assay. Dispersed human islet cells were seeded in eight-well chamber slides with harmine, DMSO or virus transduction as described above. The cells were treated with harmine or adenovirus for 72-96 hours before being fixed in 4% paraformaldehyde for 10 min, washed with PBS, and permeabilized for 10 min using 0.5% Triton-X100. The proximity of p130 to LIN52, or HA (HA-p130) to V5 (V5-WTor Mut-LIN52) was assessed using the Duolink PLA In Situ Orange Starter Kit (Mouse/Rabbit) (Sigma-Aldrich, St. Louis, MO, USA) according to the manufacturer's protocol. Blocking solution provided with the kit was added and slides were incubated in a humidified chamber at 37°C for 60 min. Slides were incubated with polyclonal rabbit LIN52 antibody (1:100) and monoclonal mouse p130 antibody (1:100) or monoclonal rabbit anti-HA (Santa Cruz Sc-805) (1:500) and monoclonal mouse V5 (Life Technologies) (1:500) overnight at 4 °C. Slides were then washed twice for 5 min in 1x DuoLink Wash Buffer A at room temperature, followed by incubation with Duolink PLA PLUS and MINUS probes diluted 1:5 in Duolink Antibody Diluent at 37 °C for 1 h. Slides were then washed with 5x Duolink Ligation Buffer diluted 1-to-5 in high-purity water, followed by incubation in Ligation Buffer for 60 min at 37 °C. All subsequent steps were performed in the dark. The 5x amplification buffer was diluted 1:5 in high-purity water, and slides were washed as above. DuoLink Polymerase was added to diluted Amplification Buffer (1:80) and slides were incubated at 37 °C for 100 min. Slides were washed twice for 10 min with 1x DuoLink Wash Buffer B. Finally, the cells were immunolabeled for C-peptide or alexa fluor-conjugated anti-glucagon (R&D Systems IC 1249C) as described above. Cover slips were mounted on slides using Duolink In Situ Mounting Medium with DAPI and sealed. Fluorescent images were captured using a Leica SP5 DMI microscope. Figures are representative of three independent experiments. Fluorescence intensity was measured using ImageJ. A minimum for 300 cells was measured for each treatment.

qPCR Methods. RNA was isolated and quantitative RT-PCR was performed as described previously (8). Gene expression in dispersed islets was analyzed by real-time PCR performed on an QuantStudio System. Primers are listed in **Suppl. Table 5**.

qPCR-ChIP Methods. p130 ChIP was performed using the EZ-ChIP Kit (Millipore) according to manufacturer's protocol. Experimental repeats (N) are indicated for each primer set in **Fig. 5**. Human cadaveric islets (500-700 IEQs, or 500,000-700,000 cells) were used per experiment for each p130 immunoprecipitation {#SC-317 (20) Santa Cruz}. Briefly, chromatin preparations were prepared using according to the EZ-ChIP Kit protocol. Primer sets were designed in the putative promoter regions of all genes tested. GAPDHS and MYBL2 primer sets have previously been published (39). Immunoprecipitated DNA was quantified using QuantStudio 5 real-time quantitative PCR detection system (Applied Biosystems). Data are presented as fold-enrichment of ChIP signal over the IgG signal. GAPDHS was used as a negative control. Primers used were:

	**Forward**	**Reverse**
GAPDHS	5'- AGACCAGCCTGAGCAAAAGA -3'	5'- CTAGGCTGGAGTGCAGTGGT -3'
MYBL2	5'- GGTGCGGCTCTAGGGA -3'	5'- CGCTACTTCGGAGTTGTGGA -3'
FOXM1	5'- GCTGCTAGACGCCCTGAC -3'	5'- TTCGGAGCTACGGCCTAA -3'
CDC25A	5'- TCTCTCCTTACCTGCCCAGA -3'	5'- GTCGTGAAGGCGCTATTTG -3'

Bioinformatics and Transcription Factor Enrichment Analysis. The Bisque4 insulinoma module genes and genes co-correlated with the module eigenvector of Bisque4 (i.e. genes reflecting module membership) at $P<0.01$ (total 253 genes, **Suppl. Table 1** and Wang et al (46) were queried in the iRegulon app (version 1.3)(50) within

Cytoscape (version 3.7.2)(63). The iRegulon options used included: Motif collection (10K 9713 PWMs); track collection (750K ChIP-seq tracks-ENCODE uniform signals) and putative regulatory region of 500bp centered around transcription start site. The enrichment score threshold was 3.0 and the maximum FDR on motif similarity was 0.001. Predicted direct target genes governed by TP53, DREAM, MMP-FOXM1 and RB-E2F were curated from Fischer et al (39). Fisher's Exact Test was used to assess significance of the geneset enrichment with a Benjamini-Hochberg multiple test correction.

Proteomics and Mass Spectrometry. Mass spectrometry samples were prepared for relative quantification as described previously (64). TMTpro reagents were used for quantification (65,66). We fractionated the pooled, labeled peptide sample using BPRP HPLC (67) and an Agilent 1200 pump equipped with a degasser and a UV detector (set at 220 and 280 nm wavelength) into 96 fractions which were concatenated into 24 super-fractions. Mass spectrometric data were collected on an Orbitrap Fusion Lumos mass spectrometer coupled to a Proxeon NanoLC-1200 UHPLC. The 100 μm capillary column was packed with 35 cm of Accucore 150 resin (2.6 μm, 150Å; ThermoFisher Scientific). The scan sequence began with an MS1 spectrum (Orbitrap analysis, resolution 120,000, 350−1400 Th, automatic gain control (AGC) target 5 $x10^5$, maximum injection time 100 ms). Data were acquired ~120 minutes per fraction. MS2 analysis consisted of collision-induced dissociation (CID), quadrupole ion trap analysis, automatic gain control (AGC) 2 $x10^4$, NCE (normalized collision energy) 35, q-value 0.25, maximum injection time 120 ms), isolation window at 0.5 Th, and TopSpeed set at 3 sec. For FAIMS, the dispersion voltage (DV) was set at 5000V, the compensation voltages (CVs) used were -40V, -60V, and -80V, and the TopSpeed parameter was set at 1 sec per CV.

Database searching included all entries from the human UniProt Database (downloaded: August 2019) with a reversed database. Searches were performed using a 50-ppm precursor ion tolerance for total protein level profiling. The product ion tolerance was set to 0.9 Da. TMTpro labels on lysine residues and peptide N-termini +304.207 Da), as well as carbamidomethylation of cysteine residues (+57.021 Da) were set as static modifications, while oxidation of methionine residues (+15.995 Da) was set as a variable modification. Peptide-spectrum matches (PSMs) were adjusted to a 1% false discovery rate (FDR) (68,69). PSM filtering was performed using a linear discriminant analysis, as described previously (70) and then assembled further to a final protein-level FDR of 1% (68). Proteins were quantified by summing reporter ion counts across all matching PSMs, also as described previously (71). Reporter ion intensities were adjusted to correct for the isotopic impurities of the different TMTpro reagents according to manufacturer specifications. The signal-to-noise (S/N) measurements of peptides assigned to each protein were summed and these values were normalized so that the sum of the signal for all proteins in each channel was equivalent to account for equal protein loading. Finally, each protein abundance measurement was scaled, such that the summed signal-to-noise for that protein across all channels equals 100, thereby generating a relative abundance (RA) measurement.

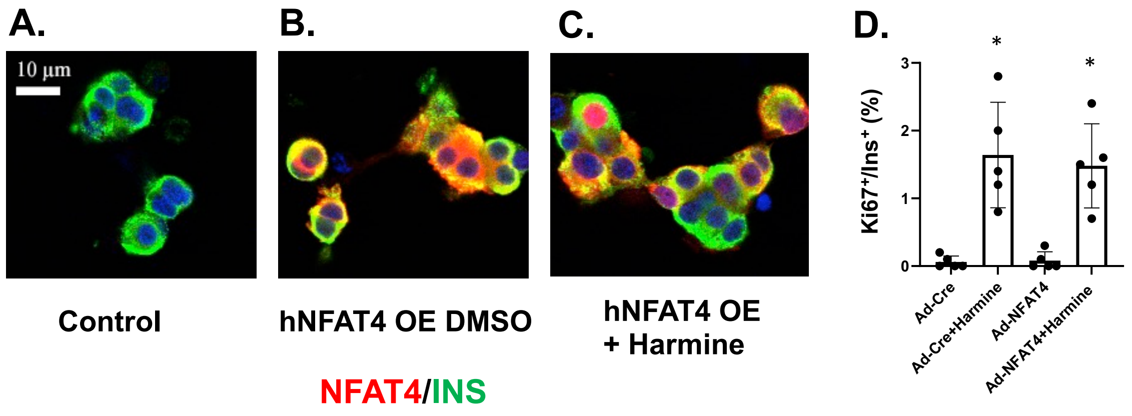

Supplemental Figure 1. Effect of Harmine on Nuclear Abundance of hNFAT4 and Beta Cell Proliferation. A. Confocal images of immunohistochemistry for endogenous hNFAT4 in human beta cells transduced with a control adenovirus expressing Cre recombinase. hNFAT4 is not visible since laser intensity is reduced to permit visualization of panels B and C. **B.** Effect of overexpression (200 moi) of wild-type hNFAT4 in human beta cells. Note that hNFAT4 is overexpressed, but limited to the cytoplasm. **C.** Overexpression of hNFAT4 in the presence of 10 uM harmine. Note that hNFAT4 is present in the nuclei of occasional beta cells. **D.** Quantification of Ki67-labeled beta cells under the conditions shown. Note that Ad.hNFAT4 does not increase Ki67 immunolabeling, while harmine does increase beta cell Ki67 immunolabeling, yet this is not further augmented by overexpression of hNFAT4. N = 5 different human islet preparations. * indicates <0.05 for Ad.Cre plus harmine and Ad.NFAT4 vs. Ad.Cre alone. Ad.NFAT4 is not significant vs. control, and Ad.Cre plus harmine and Ad.NFAT4 plus harmine are not significantly different. The white scale bar in panel C indicates 10μm.

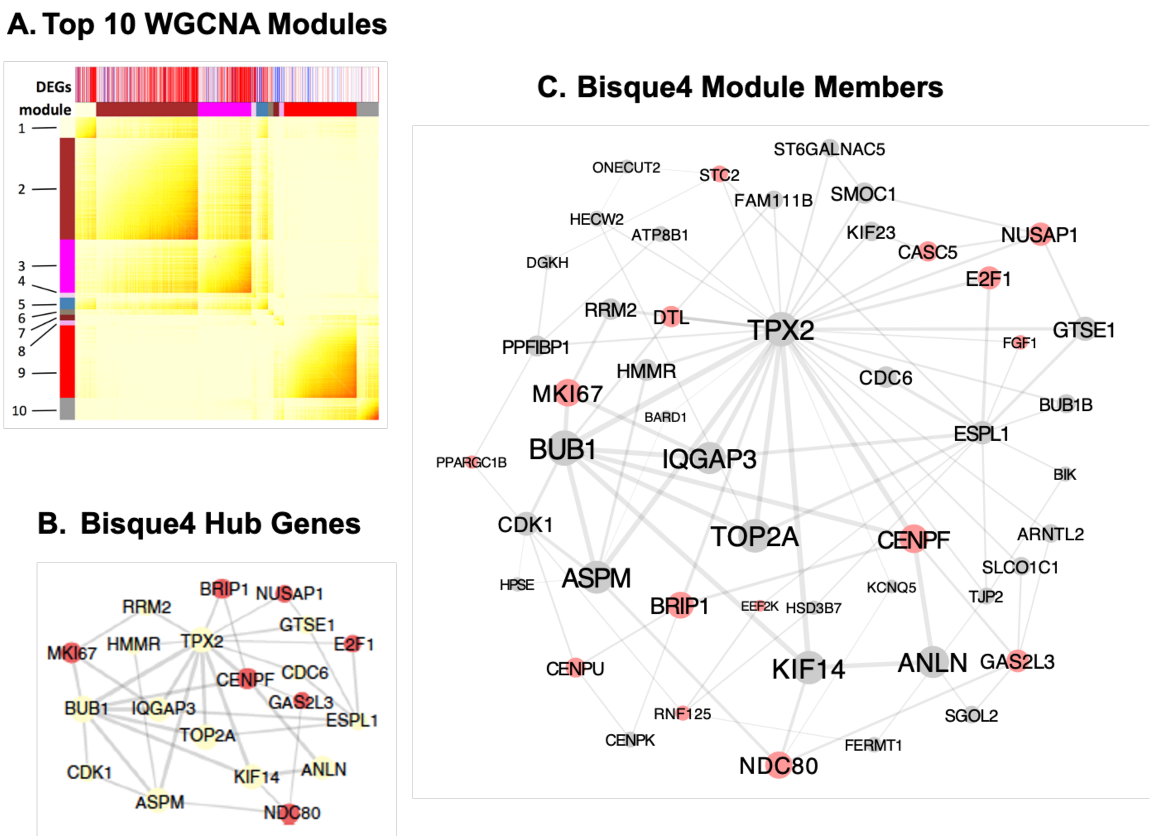

Supplemental Figure 2. Weighted Gene Co-Expression Analysis (WGCNA) of Human Beta Cells vs. Human Insulinomas Nominates the Bisque4 Cell Cycle Module as a Driver of Beta Cell Proliferation in Human Insulinoma. A. Weighted Gene Co-expression Network Analysis (WGCNA) of the transcriptomes of the 25 insulinomas vs. 22 sets of normal beta cells identified 52 co-expression modules (46), the top 10 of which are shown here. Of the 52 WGCNA modules, the Bisque4 module was the #1 module in terms of enrichment for: 1) Differentially Expressed Genes (DEGs) in insulinomas vs. beta cells; and, 2) for GO and KEGG pathway annotations related to cell cycle (46). **B.** The top 20 Bisque4 Hub genes, based on molecular connectivity (45), were genes involved in cell cycle progression (e.g., E2F1, CDK1) (46). **C.** Since the Hub and Bisque4 modules were enriched for genes involved in later stages of the cell cycle, we expanded our search for “earlier” genes, assembling a Bisque4 module membership (Bisque4$_{expanded}$) group of predicted upstream genes, predicted to be important in driving insulinoma cell cycle progression (46). This group was further explored for upstream driver transcription factors using iRegulon, as described in the text and **Fig. 2**. Note that Panels A and B were previously published in reference 46, and are provided as background.

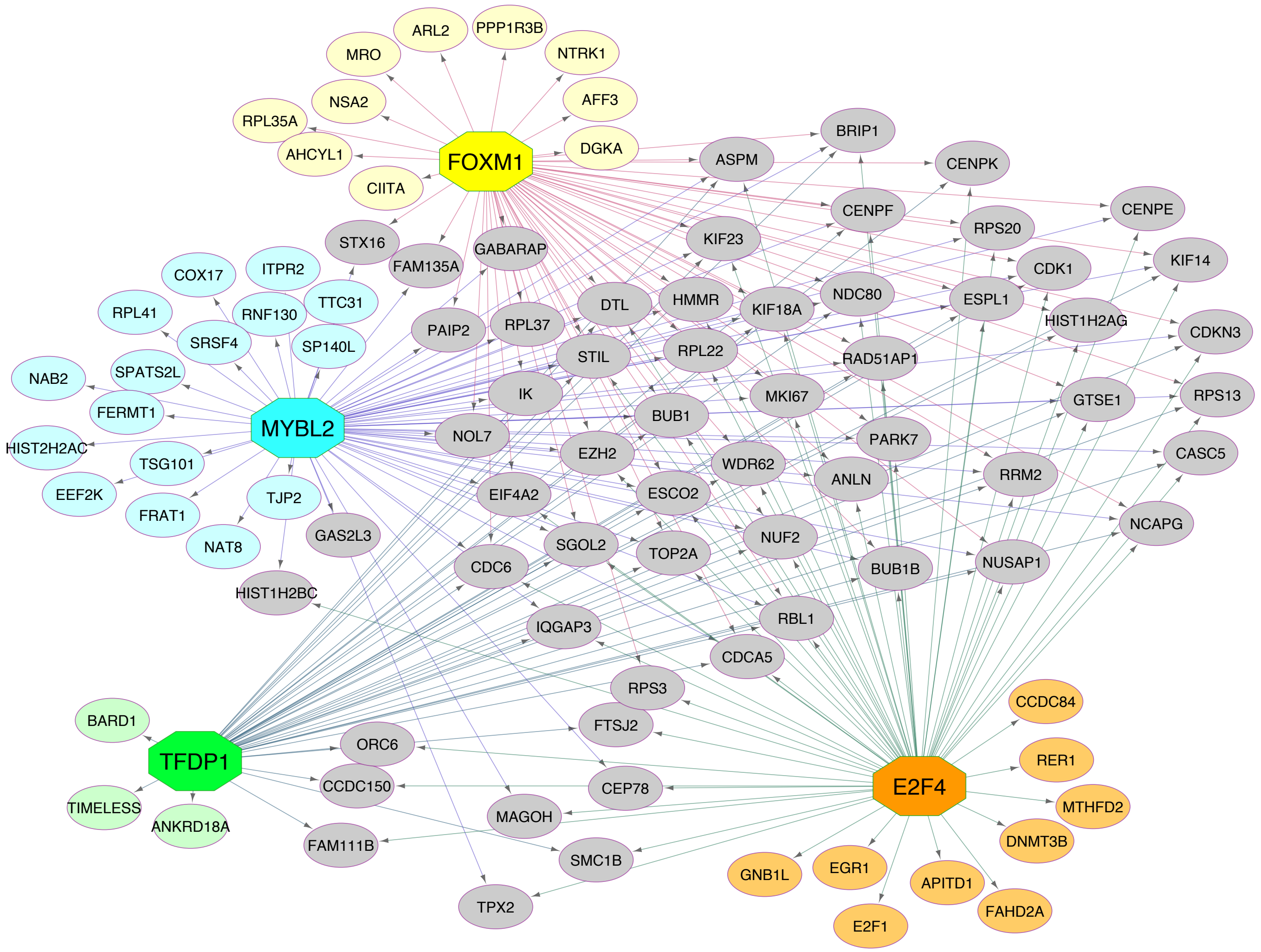

Supplemental Figure 3. Nodes and Transcription Factors Predicted by iRegulon. This is an enlarged version of **Fig. 2C** showing a network representing the predicted direct target genes governed by TFDP1, E2F4, FOXM1, and MYBL2 as determined by the iREGULON analysis. Nodes are colored according to the predicted transcriptional regulator to which they are connected in the network (yellow=FOXM1; cyan=MYBL2; green=TFDP1; orange=E2F4). Genes (nodes) which have edges/connections coming from multiple transcription factors are colored grey. See text and **Fig. 2** Legend for details.

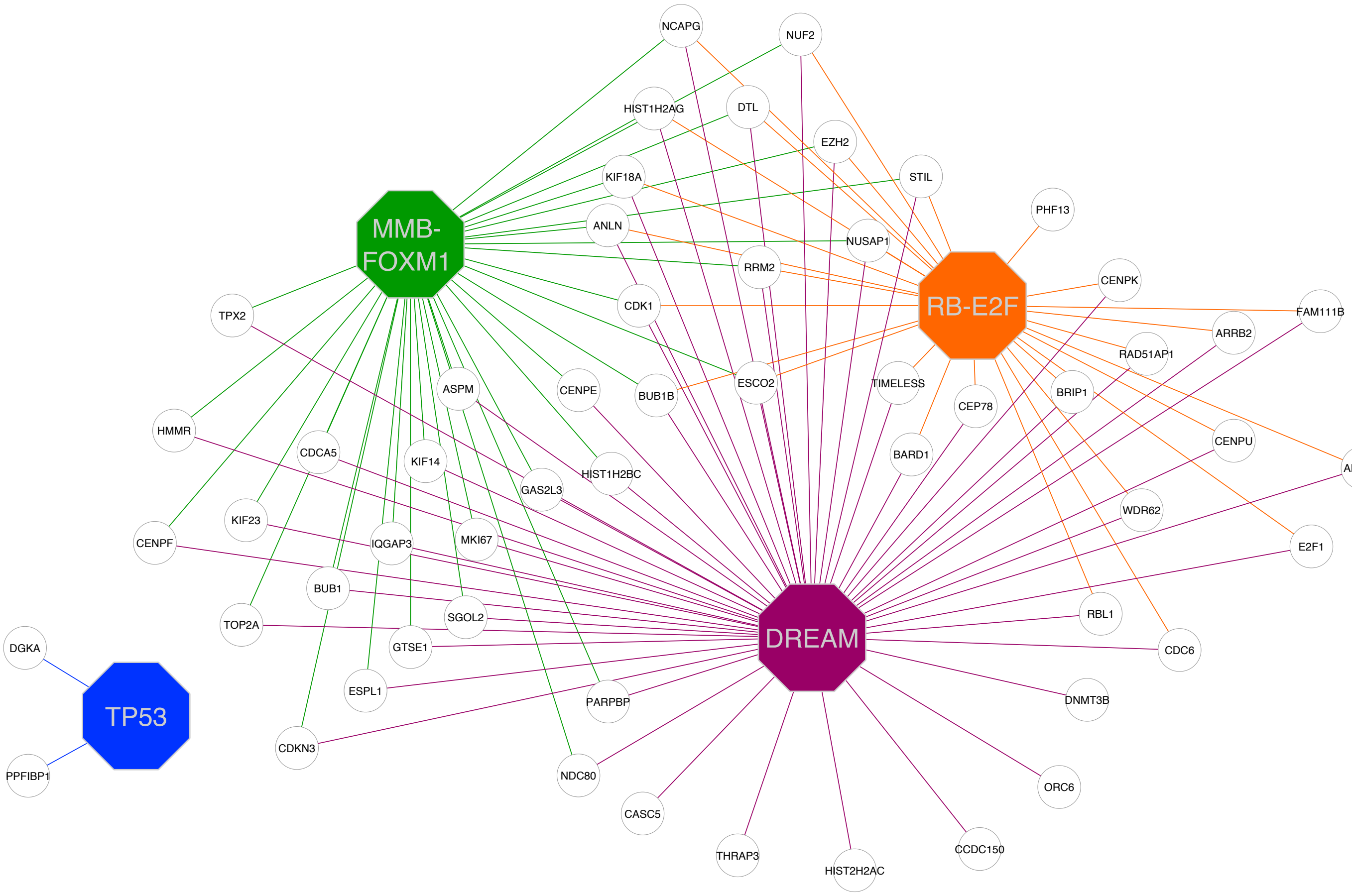

Supplemental Figure 4. Network of Predicted Targets Governed By TP53, DREAM, MMB-FOXM1 and RB-E2F (39) **That Are Also Present in the Insulinoma Bisque4-module Membership Geneset.** The network shows white nodes as the genes within the Insulinoma Bisque4-module membership geneset with colored edges connecting them to their predicted transcriptional regulators: TP53, MMB-FOXM1, RB-E2F or DREAM, as defined by Fischer et al (39). The Insulinoma Bisque4-module membership geneset was found to be significantly enriched in all but the TP53 transcription factor-targeted genes.

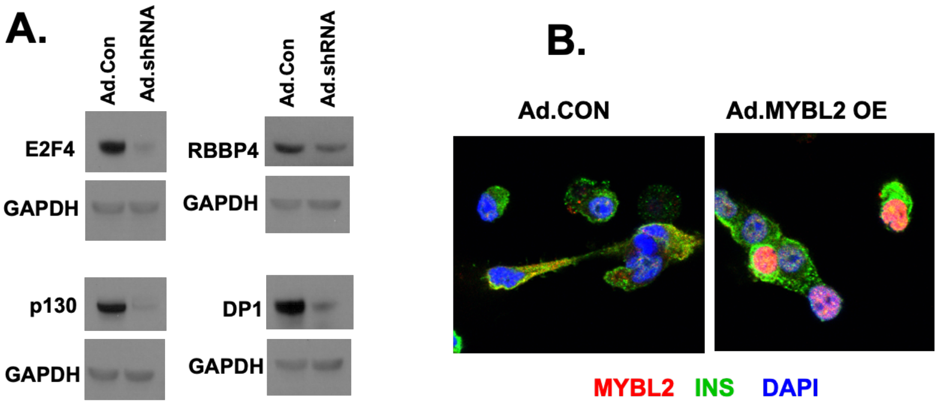

Supplemental Figure 5. Confirmation of canonical DREAM members in human islets in by immunoblot. **A.** Immunoblotting was performed on three different sets of human islet and probed with antibodies directed against E2F4, RBBP4, p130, DP1 and GAPDH as a loading control. In each case, an adenovirus expressing a corresponding shRNA was used to confirm specificity of the antibody. These results confirm and extend immunohistochemical and RNAseq findings in **Fig. 3,** and **Tables 1 and 2**. **B**. MYBL2, a target of the repressive DREAM complex, is undetectable at the immuno-histochemical level, as predicted by the DREAM model (**Fig. 2B**) and RNAseq in **Tables 1 and 2**. To confirm that the antibody used is able to detect MYBL2, we overexpressed MYBL2 by adenovirus in human islets and assessed these islets for MYBL2 expression in beta cells as shown in the far right panel. Clear MYBL2 immunolabeling is apparent. Each experiment shown is representative of experiments in three different human islet preparations.

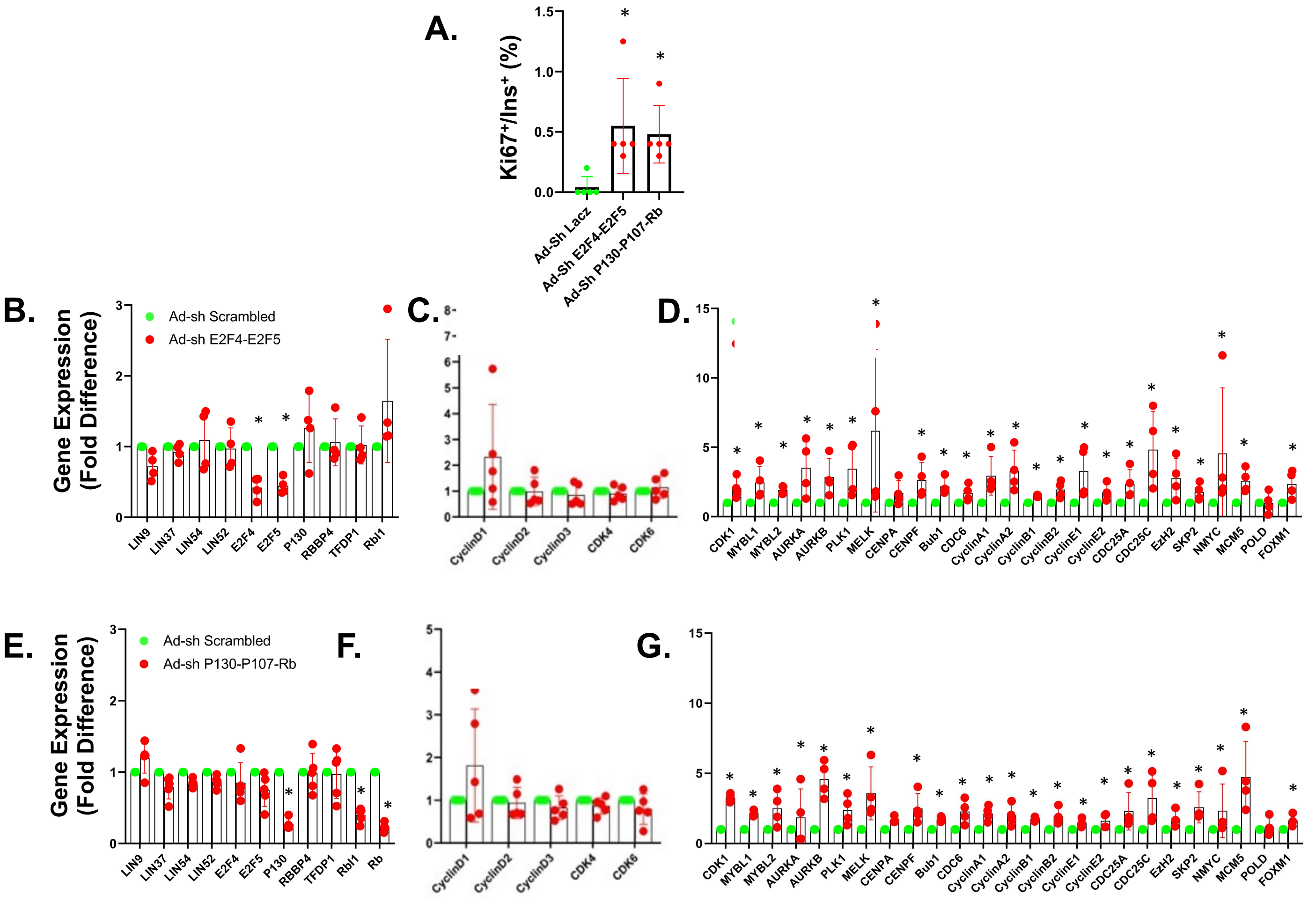

Supplemental Figure 6. Silencing E2F4 + E2F5 or all three Rb family augments beta cell proliferation, and DREAM target gene expression but not the DREAM members or the CDK4/6-Cyclin D family. A. Simultaneous silencing of E2F4 and E2F5 or pRb, p107 and p130 induces human beta cell Ki67 immunolabeling. **B-D.** qPCR analysis of human islets in which E2F4 and E2F5 were simultaneously silenced. Silencing these two DREAM members had no effect on other DREAM members or CDK4, CDK6 or D-cyclins, but led to almost uniform upregulation of canonical DREAM target genes. **E-G.** qPCR analysis of human islets in which pRb, p107 and p130 were simultaneously silenced. Again, silencing of Rb family members had no effect on DREAM members or CDK4, CDK6 or D-cyclins, but led to almost uniform upregulation of canonical DREAM target genes. Compare to **Fig. 4** and **Tables 1 and 2**. Bars indicate mean ± SEM. * indicates $p<0.05$. N = 4-5 human islet donors for all experiments.

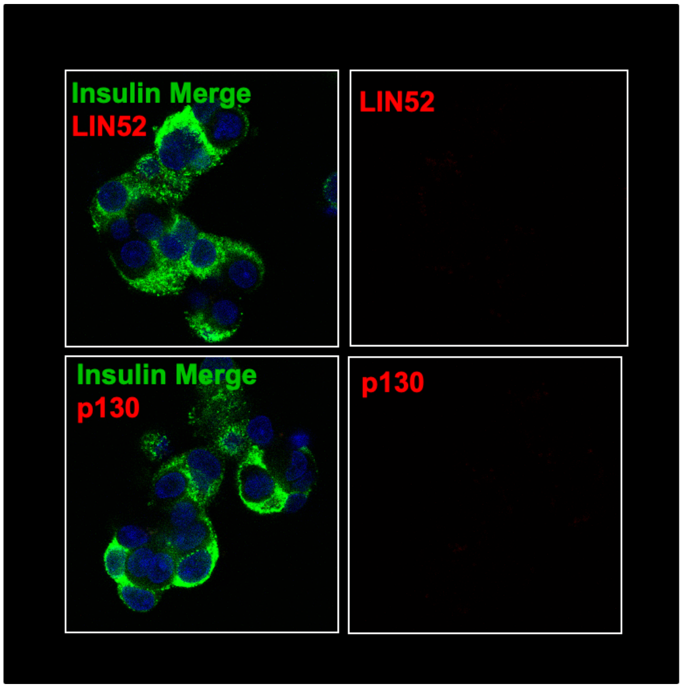

Supplemental Figure 7. A negative control for the Proximity Ligation Assay. Here, to confirm that the PLA signals in **Fig. 5A-D** are a result of proximity of LIN52 and p130, we performed simultaneous experiments in the same islets shown in Fig. 5A-D, using only single antibodies for directed against p130 or LIN52. No signal was observed, confirming the requirement for both target proteins to be present and in 40 nM proximity. Each panel is representative or 3 experiments as shown in **Figs. 5A-D.**

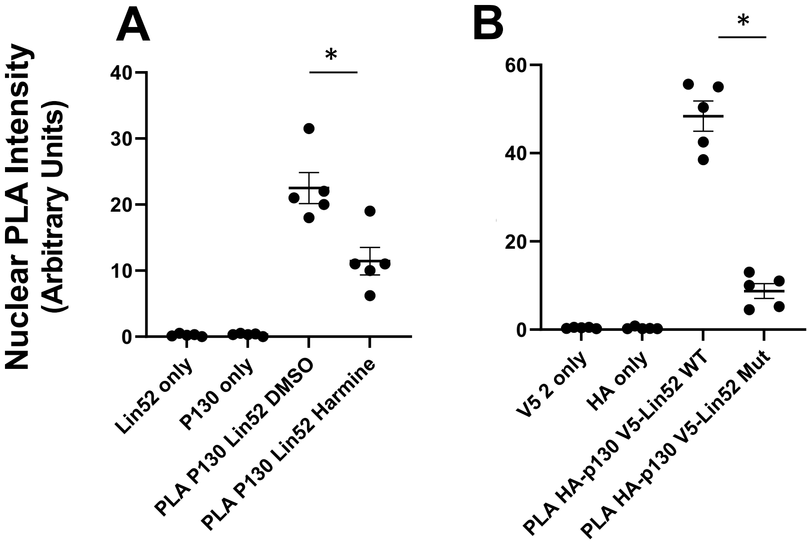

Supplemental Figure 8. Intensity of PLA fluorescence in nuclei of human insulin-positive beta cells treated with the conditions indicated. Image intensity was analyzed using ImageJ software. * indicates $p < 0.05$.

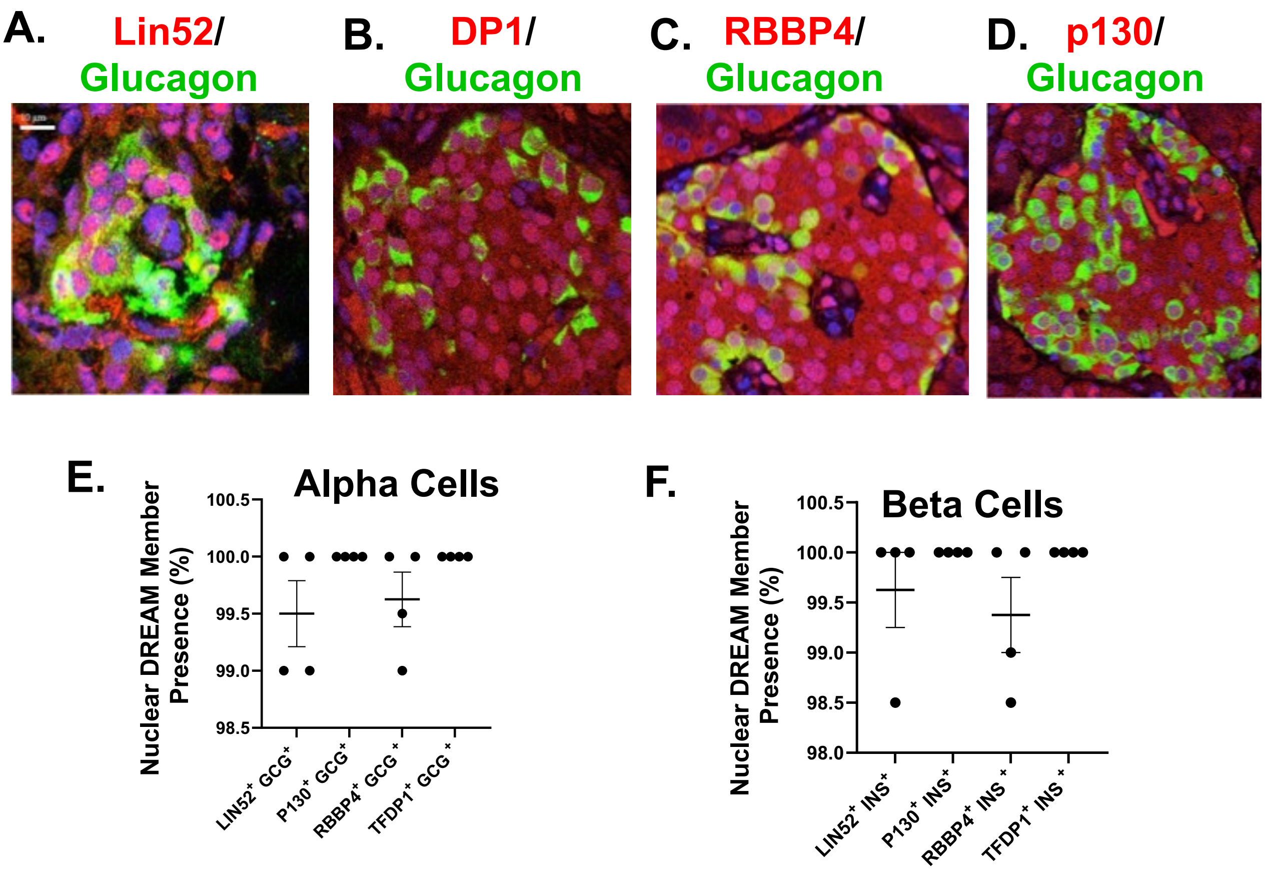

Supplemental Figure 9. DREAM members are present in human alpha cells. A-D. Glucagon and LIN52, DP1, RBBP4 and p130 co-labeling reveals that these DREAM members are present in alpha cell nuclei in normal intact human pancreas samples, as they are in beta cell nuclei in **Fig. 3C**. **E.** Essentially 100% of alpha cell nuclei contain these four DREAM members. **F.** As with alpha cells, quantification of beta cells indicates that almost every beta cell contains these three DREAM members. The white scale bar in Panel A indicates 10μm.

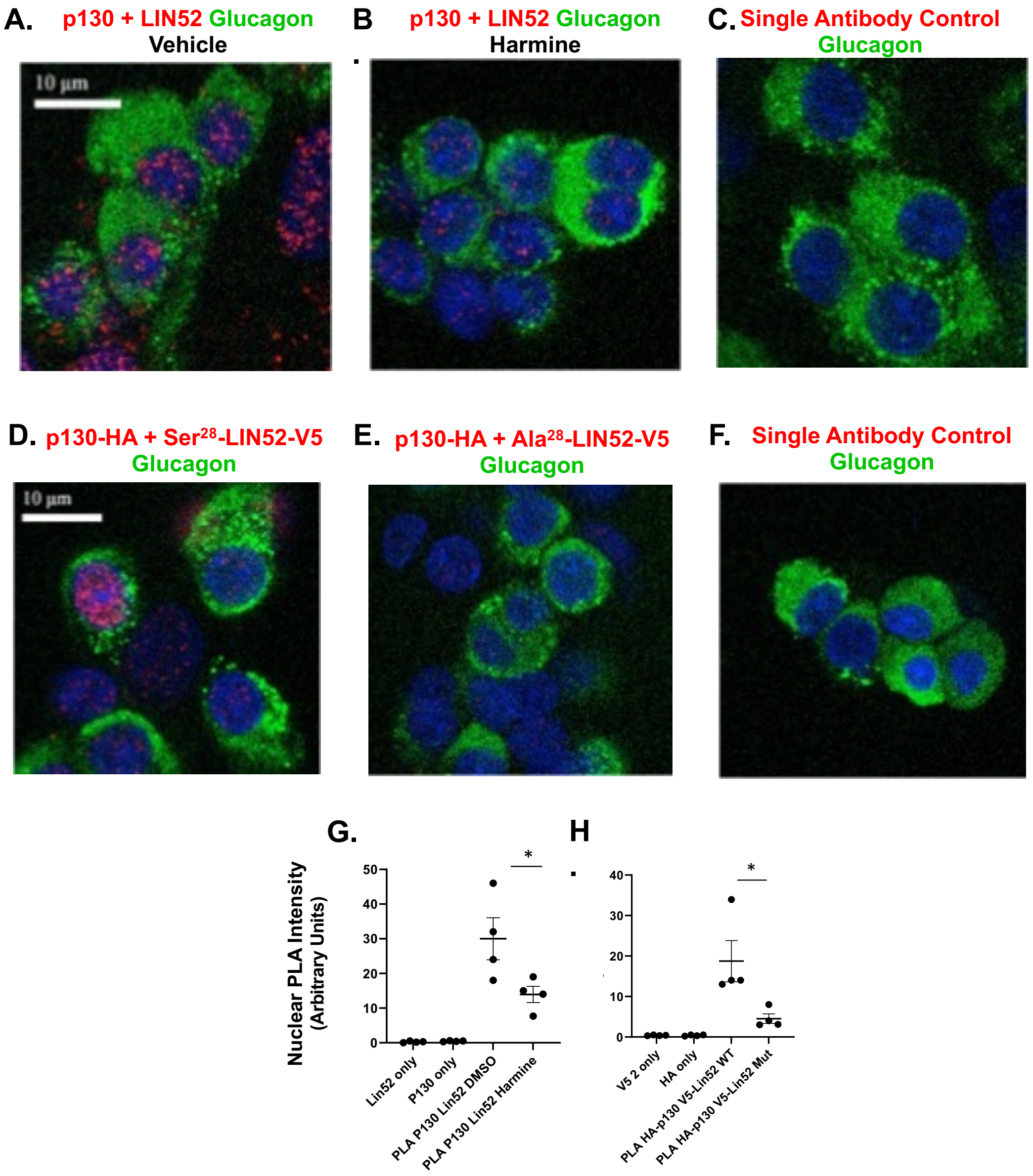

Supplemental Figure 10. Proximity ligation assay reveals interactions of DREAM members in human alpha cells, and their disruption by harmine or LIN52 Ser^{28} Mutation. A and **B.** Examples of proximity labeling of p130 and LIN52 in human alpha cells and their disruption by harmine treatment. **C.** A single antibody control (LIN-52 only) showing no proximity labeling. **D.** Proximity labeling of overexpressed LIN52-Ser^{28} with a V5 epitope tag and p130 with an HA tag. **E.** Failure of proximity labeling of LIN52 with an Ala^{28} substitution to co-localize wih p130 with an HA epitope tag. **F.** A single antibody control (LIN-52 only) showing no proximity labeling. **G. and H.** ImageJ quantification of co-localization of p130 and LIN52 under the conditions shown above. N = 4 in all experiments, * indicates $p<0.05$. The white scale bars in panels A and C indicate 10μm.

Supplemental Table 5. Primers Used for qPCR

DYRK1A forward	GGATCGTTACGAAATTGACTCCT
DRYK1A reverse	ACATAAAGTGGCGTTTCAAATGC
DYRK1B forward	ATGAACCAGCATGACACGGAG
DYRK1B reverse	CTTGAGGTCGCAGTGAATGAT
LIN9 forward	ACAGGGCTTGGAACCCATAC
LIN9 revese	TCCACGGCGACTGTCCTAAT
LIN52 forward	CTAGTTCTCCACCCAAATGGATG
LIN52 reverse	GCTGATAGGCTAGGTTCTGTAGG
LIN37 forward	AACAGCAAGAGTCGTGATGTG
LIN37 reverse	TGTTGCGATAGATGAGTGTGGA
LIN54 forward	ATTGCTAAGAAGCCTCGAACG
LIN54 reverse	TGGTGAAACTTGAGTTGTCTGTC
TFDP1 forward	AGGGCCTACGGCATTTCTC
TFDP1 reverse	CTCGTCTGCCACTTCGTTGT
RBBP4 forward	CAGCATTCATCGACTTGTCCT
RBBP4 reverse	TGTGACGCATCAAACTGAGCA
E2F4 forward	CACCACCAAGTTCGTGTCCC
E2F4 reverse	GCGTACAGCTAGGGTGTCA
E2F5 forward	ATGTCTTCTGACGTGTTTCCTC
E2F5 reverse	CGGGGTAGGAGAAAGCCTT
pRB1 forward	CTCTCGTCAGGCTTGAGTTTG
pRB1 reverse	GACATCTCATCTAGGTCAACTGC
p107 forward	CTGGACGACTTTACTGCCATC
p107 reverse	TCCAACCGTGGGAATAATGCT
p130 forward	TGAGCGAAAGCTACACGCTG
p130 reverse	TCCCTTTGCTTACAGTTGGAAC
Cyclin D1 forward	CAATGACCCCGCACGATTTC
Cyclin D1 reverse	CATGGAGGGCGGATTGGAA
Cyclin D2 forward	TTTGCCATGTACCCACCGTC
Cyclin D2 reverse	AGGGCATCACAAGTGAGCG
Cyclin D3 forward	TACCCGCCATCCATGATCG
Cyclin D3 reverse	AGGCAGTCCACTTCAGTGC
CDK4 forward	TCAGCACAGTTCGTGAGGTG
CDK4 reverse	GTCCATCAGCCGGACAACAT
CDK6 forward	CCAGATGGCTCTAACCTCAGT
CDK6 reverse	AACTTCCACGAAAAAGAGGCTT
CDK1 forward	GGATGTGCTTATGCAGGATTCC
CDK1 reverse	CATGTACTGACCAGGAGGGATAG
MYBL1 forward	GAAATCGTTGGGCAGAAATTGC
MYBL1-reverse	TGACACATACTGATACCCAGGG
MYBL2 forward	CTTGAGCGAGTCCAAAGACTG
MYBL2 reverse	AGTTGGTCAGAAGACTTCCCT
AURKA forward	GAGGTCCAAAACGTGTTCTCG
AURKA reverse	ACAGGATGAGGTACACTGGTTG
AuRKB forward	CAGTGGGACACCCGACATC
AURKB reverse	GTACACGTTTCCAAACTTGCC
PLK1 forward	CACCAGCACGTCGTAGGATTC
PLK1 reverse	CCGTAGGTAGTATCGGGCCTC
MELK forward	TATTCACCTCGATGATGATTGCG
MELK reverse	AGAAAGCCTTAAACGAACTGGTT
CENPA forward	TTCCTCCCATCAACACAGTCG
CENPA reverse	CACACCACGAGTGAATTTAACAC
CENPF forward	ACCTTCACAACGTGTTAGACAG
CENPF reverse	CTGAGGCTCTCATATTCGGCA
BUB1 forward	AGCCCAGACAGTAACAGACTC
BUB1 reverse	GTTGGCAACCTTATGTGTTTCAC
CDC6 forward	TGTTCTCCTCGTGTAAAAGCC
CDC6 reverse	GGGGAGTGTTGCATAGGTTGT
Cyclin A1 forward	GAGGTCCCGATGCTTGTCAG

Cyclin A1 reverse	GTTAGCAGCCCTAGCACTGTC
Cyclin A2 forward	GGATGGTAGTTTTGAGTCACCAC
Cyclin A2 reverse	CACGAGGATAGCTCTCATACTGT
Cyclin B1 forward	AATAAGGCGAAGATCAACATGGC
Cyclin B1 reverse	TTTGTTACCAATGTCCCCAAGAG
Cyclin B2 forward	TTGGCTGGTACAAGTCCACTC
Cyclin B2 reverse	TGGGAACTGGTATAAGCATTGTC
Cyclin E1 forward	ACTCAACGTGCAAGCCTCG
Cyclin E1 reverse	GCTCAAGAAAGTGCTGATCCC
Cyclin E2 forward	TCAAGACGAAGTAGCCGTTTAC
Cyclin E2 reverse	TGACATCCTGGGTAGTTTTCCTC
CDC25A forward	GTGAAGGCGCTATTTGGCG
CDC25A reverse	TGGTTGCTCATAATCACTGCC
CDC25C forward	ATGACAATGGAAACTTGGTGGAC
CDC25C reverse	GGAGCGATATAGGCCACTTCTG
Ezh2 forward	GTACACGGGGATAGAGAATGTGG
Ezh2 reverse	GGTGGGCGGCTTTCTTTATCA
SKP2 forward	ATGCCCCAATCTTGTCCATCT
SKP2 reverse	CACCGACTGAGTGATAGGTGT
NMYC forward	CACGTCCGCTCAAGAGTGTC
NMYC reverse	GTTTCTGCGACGCTCACTGT
MCM5 forward	GGAAGTGCAACACAGATCAGG
MCM5 reverse	AGGGACGACCTTGTCACACA
POLD1 forward	AGCAGGTCAAGGTCGTATCC
POLD1 reverse	AGCGTGGTGTAACACAGGTTG
FOXM1 forward	ATACGTGGATTGAGGACCACT
FOXM1 reverse	TCCAATGTCAAGTAGCGGTTG
